# Reduced inflammatory and Th1 transcriptional profiles in geriatric versus adult cotton rats infected with respiratory syncytial virus

**DOI:** 10.1371/journal.ppat.1014323

**Published:** 2026-07-09

**Authors:** Jonathan Miller, Cameron Leedale, Ayse Selen Yilmaz, Emily Pawlack, Jèssica Gómez Garrido, Tyler Alioto, Lianbo Yu, David E. Symer, Stefan Niewiesk

**Affiliations:** 1 Department of Veterinary Biosciences, College of Veterinary Medicine, The Ohio State University, Columbus, Ohio United States of America; 2 Department of Veterinary Microbiology and Pathology, College of Veterinary Medicine, Washington State University, Pullman, Washington United States of America; 3 Biomedical Informatics, College of Medicine, The Ohio State University, Columbus, Ohio United States of America; 4 Centro Nacional de Análisis Genómico (CNAG), Barcelona, Spain; 5 Universitat de Barcelona (UB), Barcelona, Spain; 6 Division of Hematology and Oncology, Department of Medicine, VA Boston Healthcare System, US Department of Veterans Affairs, Boston, Massachusetts United States of America; Wageningen Bioveterinary Research, NETHERLANDS, KINGDOM OF THE

## Abstract

Human respiratory syncytial virus (RSV) is a leading cause of severe respiratory disease in elderly populations. RSV clearance is delayed in individuals over 65 years of age, a finding that is recapitulated in the geriatric cotton rat model. Altered inflammatory responses in elderly populations have been implicated with impaired clearance of several pathogens but remain poorly described for RSV. We performed RNA sequencing (RNA-seq) and pathway analysis on lung tissue of RSV-infected adult and geriatric cotton rats to characterize differential host immune responses between these age groups. No differences in inflammatory gene expression were observed between uninfected adult and geriatric cotton rats. At day 1 post-infection, adult cotton rats expressed higher levels of genes encoding several cytokines and transcription factors associated with acute inflammation and initiation of a Th1 response, including IFN- β, IL12, and Tbet, compared to geriatric cotton rats. By day 4 post-infection, adult cotton rats additionally expressed higher levels of genes encoding effector molecules associated with Th1 responses, including IFN-γ. Pathway analysis showed significantly increased activation of several pro-inflammatory pathways in adults, including the Th1 pathway, macrophage classical (M1) activation, and pathogen-induced cytokine signaling. To evaluate lymphocyte Th1/Th2 polarization resulting from RSV infection in both age groups, we stimulated splenocytes with RSV antigen at day 28 post-infection and measured IFN-γ and IL-4 production by ELISA. Splenocytes of all adult cotton rats produced detectable IFN-γ and no IL-4, indicating Th1 polarization. Splenocytes of all geriatric cotton rats produced detectable IL-4, indicating Th2 polarization. Collectively, these results indicate that geriatric cotton rats exhibit impaired generation of a Th1 response and a decreased inflammatory response overall to RSV.

## Author summary

Respiratory syncytial virus (RSV) is one of the leading causes of respiratory illness in humans worldwide and is of particular importance in elderly people. In individuals over 65 years of age, immune responses against RSV are less effective than in younger people, resulting in delayed clearance of the virus and increased rates of severe disease. To better understand how immune responses between elderly and younger populations may differ during the course of RSV infection, we gene expression in the lungs of geriatric and young adult cotton rats over several days of experimental infection. Cotton rats, the preferred small animal model for studying natural RSV infection, demonstrated significantly lower pro-inflammatory expression in the geriatric group, as measured by individual gene expression and pathway analysis. A prominent example in the geriatric cotton rats was decreased expression of the Th1 pathway, an immune response that is highly associated with effective antiviral immunity, with decreased production of key molecules such as the effector cytokine IFN-γ.

## Introduction

Respiratory syncytial virus (RSV) is a common respiratory infection throughout all age groups but is associated with disproportionately severe clinical consequences in high-risk groups including older individuals. In healthy adults, RSV infection typically results in mild respiratory signs resulting from replication and concomitant inflammation in the upper airways. In the elderly, the risk of more severe and prolonged disease resulting from RSV infection rises significantly. Among adults in the United States over 65 years of age, an estimated 60,000–160,000 hospitalizations and 6,000 – 10,000 deaths are attributed to RSV annually [1–4]. The true rate of hospitalizations and deaths resulting from RSV infection may be higher than reported, given that lower respiratory tract infection by RSV results in similar clinical signs as those caused by other respiratory viruses such as influenza [1,5]. Underlying chronic conditions and comorbidities including chronic obstructive pulmonary disease, diseases of immunosuppression, cystic fibrosis, and congestive heart failure are experienced at increased rates in the elderly, which can contribute to the severity of RSV-associated disease. However, elevated rates of these predisposing conditions do not fully account for the marked increase in severe clinical disease among RSV-infected elderly individuals, indicating that other age-associated factors contribute to susceptibility in this age group [6].

The increase in severity of respiratory infections in the elderly has been linked to numerous age-associated declines in innate and adaptive immunity, commonly referred to as immunosenescence [7–9]. Tissue level changes include thymic involution, resulting in reduced output of naïve T cells, and decreased germinal center differentiation, resulting in impaired antigen-specific B cell expansion [10–12]. Detrimental cellular changes associated with immunosenescence have been described in most leukocyte compartments. Neutrophils show decreased superoxide production and phagocytic activity, while aberrant chemotaxis results in poorly targeted and inappropriate inflammation [13–16]. Mononuclear phagocytes of aged individuals exhibit diminished antigen-presentation function upon stimulation and decreased responsiveness to signaling through pathogen pattern recognition receptors such as Toll-like receptors [17–20]. Stimulated natural killer cells of aged individuals and animal models demonstrate impaired cytotoxicity and produce reduced IFN-γ and TNF-α, potentially contributing to decreased responsiveness of T cells and dendritic cells [21–25]. B cells produce a less diverse antibody repertoire with age, while impaired class-switching and somatic recombination reduce the average affinity of antibodies and contribute to lower neutralizing antibody titers upon natural infection or vaccination [12,26–29]. T cell receptor diversity is decreased with age, in part due to the aforementioned atrophy of the thymus throughout life [30,31]. Increased expression of inhibitory receptors and decreased expression of costimulatory molecules is a hallmark of senescent T cells [32]. CD8 + T cells also produce lower levels of cytotoxic molecules such as perforins and granzymes. These perturbations and deficiencies across innate and adaptive immune compartments have been shown to play a role in susceptibility to a wide array of infectious and autoimmune diseases in the elderly. However, the relative importance of each immunosenescence-associated change varies by condition. The specific factors that lead to prolonged and enhanced disease in RSV-infected elderly individuals are incompletely characterized.

Animal models of RSV infection have shed some light on host immunological deficiencies in aging. Aged mice exhibit delayed RSV clearance, similar to kinetics observed in elderly humans, that correlate with impaired CD8 + T cell responses relative to young animals [33,34]. Cotton rats, the preferred small animal model for testing and developing therapeutics and vaccines for RSV, also demonstrate delayed RSV clearance and CD8 + T cell responses [35–38]. Cotton rats have a significantly shorter lifespan than mice, facilitating their use for studies using geriatric cohorts, and allow for productive RSV infection with replication of approximately 100-fold higher titer than seen in BALB/C mice [39]. In both aged mice and cotton rats, impaired generation of a CD8 + T cell response to RSV has been linked to decreased pulmonary dendritic cell maturation and migration to regional lymph nodes [38,40]. Humans and both animal models also show reduced humoral immune responses to RSV in aged cohorts. Decreased neutralizing antibody titers correlate with more severe clinical disease in elderly people and reduced duration of protective immunity in old mice and cotton rats [37,41,42]. The underlying basis of these diminished or delayed effector responses remains largely unexplored, although a few alterations in cytokine expression profiles of geriatric humans and animal models have been described. In individuals over 65 years of age, peripheral blood mononuclear cells (PBMCs) produce lower levels of several proinflammatory and antiviral cytokines and chemokines upon activation, including IFN-γ, type I IFNs, TNF-α, IL-6, CCL2, and CCL7 [43,44]. Among individuals over 75 years of age, PBMCs co-cultured with autologous dendritic cells and stimulated with RSV also produce lower levels of IFN-γ compared to younger cohorts [45]. Measurement of expression of select cytokines and chemokines in young and geriatric cotton rats infected with RSV found delayed peaks in expression of IFN-γ, IL-6, and IL-10 in older cohorts [35].

One throughline of these findings is a reduction in IFN-γ production in RSV-infected geriatric individuals and animals compared to young cohorts. IFN-γ is a critical propagator and effector molecule in Th1 responses, and its expression is strongly correlated with clearance of a variety of respiratory viruses including RSV [46–49]. More generally, these previous surveys of select cytokines and chemokines show a trend of decreased production of pro-inflammatory factors in the elderly. To investigate whether this trend of depressed inflammation would hold true across the lung transcriptome during RSV infection, we performed mRNA sequencing (RNA-seq) on lungs of young adult and geriatric cotton rats at various timepoints post-infection with RSV. We then performed pathway analysis of differentially expressed genes between the two age groups to investigate broader trends in inflammatory pathways during infection. To corroborate the magnitude of differentially expressed genes, we also performed digital droplet polymerase chain reaction (ddPCR) on lung samples for select inflammatory factors shown to be upregulated during RSV infection. Finally, we measured IFN-γ and IL-4 protein expression by splenocytes of previously infected cotton rats upon stimulation with RSV antigen as a measure of Th1 and Th2 differentiation.

## Methods

### Ethics Statement

All animal studies received approval by the Institutional Animal Care and Use Committee of The Ohio State University.

### Animals

Inbred cotton rats (*Sigmodon hispidus*) were purchased from Inotiv (formerly Envigo; West Lafayette, IN). All cotton rats were maintained in NexGen Rat 900 polysulfone microisolator cages (Allentown Inc., Allentown, NJ) in a barrier facility with 12:12 hour light cycles, 22 ± 2 °C, and 20–70% relative humidity. Age ranges were 1–4 months for adult animals and >8 months for geriatric animals [35–38]. For mRNA-seq and qPCR experiments, adult groups were composed of 3 female and 3 male cotton rats, and geriatric groups were composed of 4 female and 2 male cotton rats. All studies received approval by the Institutional Animal Care and Use Committee of The Ohio State University.

### Virus

RSV-A2 (NCBI taxonomy ID: 11259) stocks were propagated in HEp-2 cells in MEM (Thermo Fisher Scientific, Rockford, IL) supplemented with 2% fetal bovine serum and stored in liquid nitrogen as previously outlined [50]. Titers of RSV stocks were quantified by standard TCID_50_ assay, summarized briefly as follows. HEp-2 cells were seeded at 80% confluency on 48-well plates. 10-fold serial dilutions of RSV stocks were plated onto the cells with 500 µL MEM for one hour at 37°C and 5% CO_2_, using 6 wells per concentration. Cells were washed three times with PBS/0.1% FCS, after which 500 µL MEM/2% FCS was added to each well. Cells were incubated at 37°C and 5% CO_2_. Media was replaced on day 3. All wells were evaluated for viral cytopathic effect by light microscopy following 5 total days of incubation. Viral titer was calculated using the Reed-Muench method and expressed as the quantity of virus capable of infecting 50% of inoculated tissue culture monolayers (TCID_50_) [51].

### Cotton rat infection and tissue collection

Cotton rats were anesthetized via isoflurane inhalation. RSV-A2 stocks were diluted in PBS and 10^5^ TCID_50_ was administered intranasally in an inoculation volume of 100 µL. At indicated end-points, cotton rats were euthanized by CO_2_-induced asphyxiation followed by exsanguination. For RNA-seq and qPCR experiments, the left lung lobe was collected and placed in 2 mL RNAprotect (QIAGEN, Hilden, Germany) at room temperature. For splenocyte stimulation experiments, the spleen was collected and placed in 10 mL PBS on ice.

### RNA extraction

Following euthanasia, lungs were placed in RNAprotect (QIAGEN) at room temperature for 30 minutes. 25–30 µg of each lung sample was removed by sterile dissection and placed in 2 mL Precellys bead tubes (Bertin Technologies, France) with 600 µL Buffer RLT Plus (QIAGEN) and 6 µL β-mercaptoethanol. Samples were homogenized at room temperature with a Precellys 24 tissue homogenizer (Bertin) at 6000 RPM, 3 cycles for 30 seconds each with 10-second breaks. The homogenate was passed through QIAshredder tubes (QIAGEN). RNA was extracted and isolated with RNeasy Plus Mini extraction kits (QIAGEN) following manufacturer instructions, with a final elution volume of 30 µL. RNA concentrations, 260/280 ratios, and 260/230 ratios were measured using a NanoDrop One spectrophotometer (Thermo Fisher), ensuring a minimum concentration of 100 ng/µL and 260/280 ratios between 1.8 and 2.2 for all extracted RNA samples.

### Library preparation and mRNA-seq

RNAseq of all experimental samples was performed through the Genomics Shared Resource at the Ohio State University Comprehensive Cancer Center. The RNA Integrity Number (RIN) for DNase-treated total RNA were assessed using Agilent BioAnalyzer RNA Nano Kit (Agilent Technologies, Inc., Santa Clara, CA) and the RNA amount was assayed with the Invitrogen Qubit RNA HS Assay Kit (Thermo Fisher Scientific, Waltham, MA). Samples with RIN > 7 were used in mRNA-seq library generation using the QIAseq FastSelect RNA Lib Core Kit (QIAGEN) and the QIAseq Stranded mRNA Enrichment Kit (QIAGEN). Briefly, 100 ng DNase-treated total RNA was used as input for library generation. RNA fragmentation was set for 3 minutes at 94C. 17 PCR cycles were used in final library generation. Library quantification and characterization were assessed with Agilent BioAnalyzer HS DNA Kit and the Invitrogen Qubit DNA HS Assay Kit. Libraries were pooled together with other index – compatible RNA-seq libraries for sequencing on Illumina NovaSeq X Plus 10B (Illumina Technology, Portland, OR) flow cell lane to a minimum depth of 15–20 million clusters per sample.

### Alignment and gene annotation

150 bp paired-end reads were aligned using Hisat2 version 2.1.0 [52] against the *Sigmodon hispidus* complete DNA reference assembly [53]. Alignment parameters were set with 2 mismatches permitted to allow for potential base-calling errors. Reads that aligned to multiple loci were discarded. Mapped reads were quantified using featureCounts software [54] and the *Sigmodon hispidus* annotation in gtf format. The resulting count matrix was annotated with *Sigmodon hispidus* gene IDs, associated Ensembl gene IDs, and mouse (*Mus musculus*) gene orthologs which was then used to perform the differentially expressed gene (DEG) analysis.

### Differentially expressed genes and pathway analysis

R package limma [55] was used for analysis of DEGs. Read counts were normalized using TMM method [56]. DEGs were defined as transcripts with false discovery rate-adjusted *p*-values (*q*-values) of less than 0.05 and fold change values of greater than 2 or less than 0.5. Signaling and metabolic pathway analysis of DEGs was performed using the Ingenuity Pathway Analysis (QIAGEN) software package and the Ingenuity Knowledge Base reference database (accessed August 31, 2023). Significance of alterations between groups for a given canonical pathway was calculated using a Fisher’s exact test, with p-values of ≤ 0.05 considered significant. Up/down-regulation patterns between groups for canonical pathways were assigned an activation z-score using an asymptotic Gaussian approximation [57] with |z-scores| greater than 2 considered significant for directionality.

### Digital droplet PCR

First-strand complementary DNA (cDNA) was generated with iScript cDNA Synthesis kits (Bio-Rad, Hercules, CA) following manufacturer-recommended instructions and thermal cycling conditions, using 1 µg of RNA samples extracted from whole lung as described above. cDNA concentrations were quantified with a NanoDrop One spectrophotometer (Thermo Fisher). Absolute quantification of *CXCL10* (encoding IP-10), *IL12A* (IL-12p35), *IFNG* (IFN-γ), and *CXCL1* (GRO) expression in 300 ng cDNA was measured by digital droplet polymerase chain reaction (ddPCR). Reactions were prepared with ddPCR Multiplex Supermix (Bio-Rad). All primers and probes were obtained from Integrated DNA Technologies (Coralville, IA). Primer concentrations of 900 nM and probe concentrations of 250 nM were used. For cotton rat *CXCL10* (GenBank accession AF421394.1), the forward primer sequence was CGCCCTTTAACCAGAGAGAAG, the reverse primer sequence was GTAGAGAGAAGTGGTGGAGAGA, and the probe sequence was ACAGACGAGTGGCAGAGAGCC. For cotton rat *CXCL1* (GenBank accession AF421393.1), the forward primer sequence was TCTCTGTCACTTGCTGATGC, the reverse primer sequence was AGCTTGCCTTAACCCTGAAG, and the probe sequence was TCTGGACAATCTTCTGGACCATGGG. For cotton rat *IFNG* (GenBank accession AF167349.1), the forward primer sequence was GTGCATCACTCCAACAAGTTC, the reverse primer sequence was GCAGAGAGAAGATGGATGACTT, and the probe sequence was TGCGCTGGACCTGAAGATCGTTTA. For cotton rat *IL12A* (GenBank accession AF421396.1), the forward primer sequence was GTGTCTCGCCCATTCTCAAA, the reverse primer sequence was CTTCAGCAGTGCAGGGATAAT, and the probe sequence was AACCACATGCTGGAGAAGGCCATA. Droplets were generated using a QX200 droplet generator (Bio-Rad) following manufacturer recommendations. A C1000 thermal cycler (Bio-Rad) was used for PCR amplification. Reactions began with enzyme activation step at 95°C for 10 min followed by 40 cycles with a denaturation temperature of 94°C for 30 seconds and annealing and extension at 65.6°C for 90 seconds. Reactions were terminated with an enzyme deactivation step at 98°C for 10 min. Droplet fluorescence for each gene fragment was measured with a QX600 droplet reader (Bio-Rad). QX Manager Standard Edition 2.0.0 (Bio-Rad) was used to set automatically-generated thresholds for positive events and determine the proportion of droplets with positive fluorescence. The concentration of each gene was calculated as copies/ng of cDNA. The total event count (droplet counts) for all samples was > 10,000.

### Splenocyte stimulation and cytokine ELISA

5 adult (<2 months old) and 3 geriatric (>12 months old) cotton rats were infected with RSV. On day 27 post-infection, 96-well plates were coated with 200 µL of 10 µ/mL (50 μl/well) of sucrose gradient-purified, UV-inactivated RSV-A2 antigen in 200 mM Na_2_CO_3_ buffer (pH 9.6) at 4°C overnight. Cotton rats were euthanized on day 28 post-infection for spleen collection. All adipose and connective tissue at the splenic hilum was trimmed and spleens were gently pressed through 100 µm mesh cell strainers (Fisher Scientific) to generate single-cell suspensions. Splenocytes were washed 3 times with PBS and counted by trypan blue exclusion and light microscopic examination. 5 * 10^5^ splenocytes were plated in each well of an RSV antigen-coated 96 well plate, with 4 replicates per animal. The splenocytes were incubated in 200 μL Advanced RPMI 1640 media (Thermo Fisher) with 2% cotton rat serum for 3 days. The concentration of cotton rat IFN-γ in the pooled supernatant of all replicates for each animal was measured by standard ELISA kit (R&D Systems) following manufacturer recommended protocols. The concentration of cotton rat IL-4 in the pooled supernatant of all replicates for each animal was measured with a biotinylated anti-cotton rat IL-4 antibody (R&D Systems) and following manufacturer recommended protocols, using R&D Systems Color Reagent A (H_2_O_2_) and Color Reagent B (tetramethylbenzidine) for substrate solution and 2N H_2_SO_4_ as stop solution. Both ELISAs were performed in Maxisorb (NUNC) 96-well ELISA plates, with washes between subsequent steps using 0.05% Tween 20 and blocking using 1% BSA. Optical densities of each well were measured with a Tecan microplate reader set to a target wavelength of 450 nm with subtraction of wavelength correction at 570 nm. Linear regression analysis was used to determine the concentration of samples against a standard curve run in duplicate.

## Results

### Lung transcriptome changes induced by RSV infection

Whole lung transcriptional profiles in adult and geriatric cotton rats were compared over the course of RSV infection and between age groups. Treatment groups were composed of 6 cotton rats between 1 and 4 months of age (adult) or over 8 months of age (geriatric). Samples of whole lung were obtained for RNA-seq at days 1 and 4 post-infection with RSV, or day 1 post-inoculation with PBS (uninfected). Differentially expressed genes (DEGs) were defined as genes with a fold-change (FC) in transcript counts of 2 or greater between groups and q-values of under 0.05. Pathway analyses were performed in Ingenuity Pathway Analysis based on the defined DEG thresholds, with activation |z-scores| of greater than 2 considered significant for directionality.

**Fig 1:**
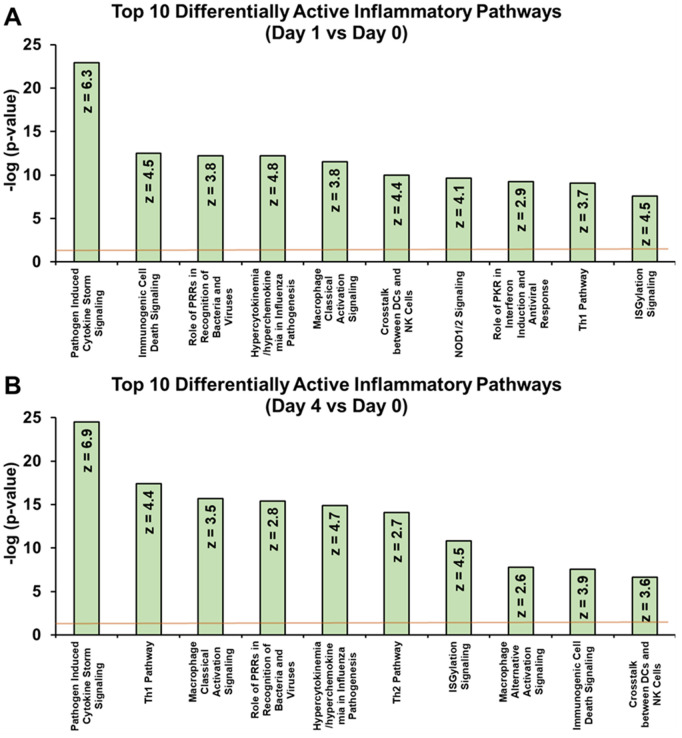
Top differentially activated inflammatory pathways in adult cotton rats at days 1 and 4 post-RSV infection. Pathway analysis was based on DEGs (*p* < 0.05, FC > 2 or <0.5) in the lung of adult cotton rats at days 1 (A) or 4 (B) post-infection with RSV compared with that of uninfected adults. The orange line represents the threshold for significance in pathway activity (*p* = 0.05, -log(*p*) = 1.3). All shown pathways on both days 1 and 4 were more active in infected animals compared to baseline (*z* > 2). (*n* = 6 for all groups).

Compared to PBS-treated (Day 0 post-infection) animals, adult cotton rats demonstrated inflammatory transcriptional profiles at days 1 and 4 post-RSV infection, as expected for respiratory viral infection. At day 1, the most differentially regulated gene was *CXCL10* (IP-10), with a FC in expression of 845 compared to uninfected cotton rats (*q* = 1.2E-12). IP-10, a chemokine associated with chemotaxis of a variety of leukocytes including dendritic cells, monocytes, natural killer (NK) cells, and T cells, has been shown to be highly upregulated both *in vitro* and *in vivo* in the early stages of RSV infection [58–60]. Other top inflammatory or antiviral DEGs at day 1 included *IFNB1* (FC = 728, *q* = 3.9E-11), *IL6* (FC = 168, *q* = 4.6E-5), *ISG15* (FC = 97, *q* = 1.2E-15), *IRF7* (FC = 91, *q* = 2.0E-13), *CCL2* (FC = 72, *q* = 2.6E-5), and *CXCL9* (FC = 71, *q* = 6.8E08). This acute inflammatory profile was reflected in the pathway analysis (Fig 1A), which demonstrates enrichment of pathways associated with inflammation (Pathogen Induced Cytokine Storm Signaling Pathway, Role of Hypercytokinemia/hyperchemokinemia in the Pathogenesis of Influenza), innate immune response (Role of Pattern Recognition Receptors (PRRs) in Recognition of Bacteria and Viruses, NOD1/2 Signaling Pathway, Role of PKR in Interferon Induction and Antiviral Response, ISGylation Signaling Pathway), and cellular immune response (Immunogenic Cell Death Signaling Pathway, Macrophage Classical Activation Signaling Pathway, Crosstalk between Dendritic Cells and Natural Killer Cells, Th1 Pathway).

At day 4, *CXCL10* remained the most highly differentially expressed gene (FC = 136, *q* = 1.02E-8) followed by *IL22RA1* (FC = 112, *q* = 1.09E-6), encoding the unique alpha subunit of the IL-22 receptor. Other top inflammatory or antiviral DEGs on day 4 included *CXCL9* (FC 41, *q* = 3.16E-6), *IRF7* (FC 30, *q* = 1.19E-9), *ISG15* (FC 26, *q* = 2.27E-11), *CCL2* (FC 23, *q* = 2.74E-3), and *OASL* (FC 22, *q* = 5.4E-11). Many of the same inflammatory and immune pathways that were elevated at day 1 remained elevated at day 4 (Fig 1B). However, the most upregulated pathways at day 4 also included notable immunomodulatory/anti-inflammatory pathways (Th2 pathway, Macrophage Alternative Activation Signaling Pathway) and specifically the gene encoding the anti-inflammatory cytokine IL-10 which has been shown to have protective roles during later stages of RSV infection in humans by regulating excessive inflammation and reducing tissue injury [61–63].

### Pro-inflammatory pathways are less active in RSV-infected geriatric cotton rats

**Fig 2:**
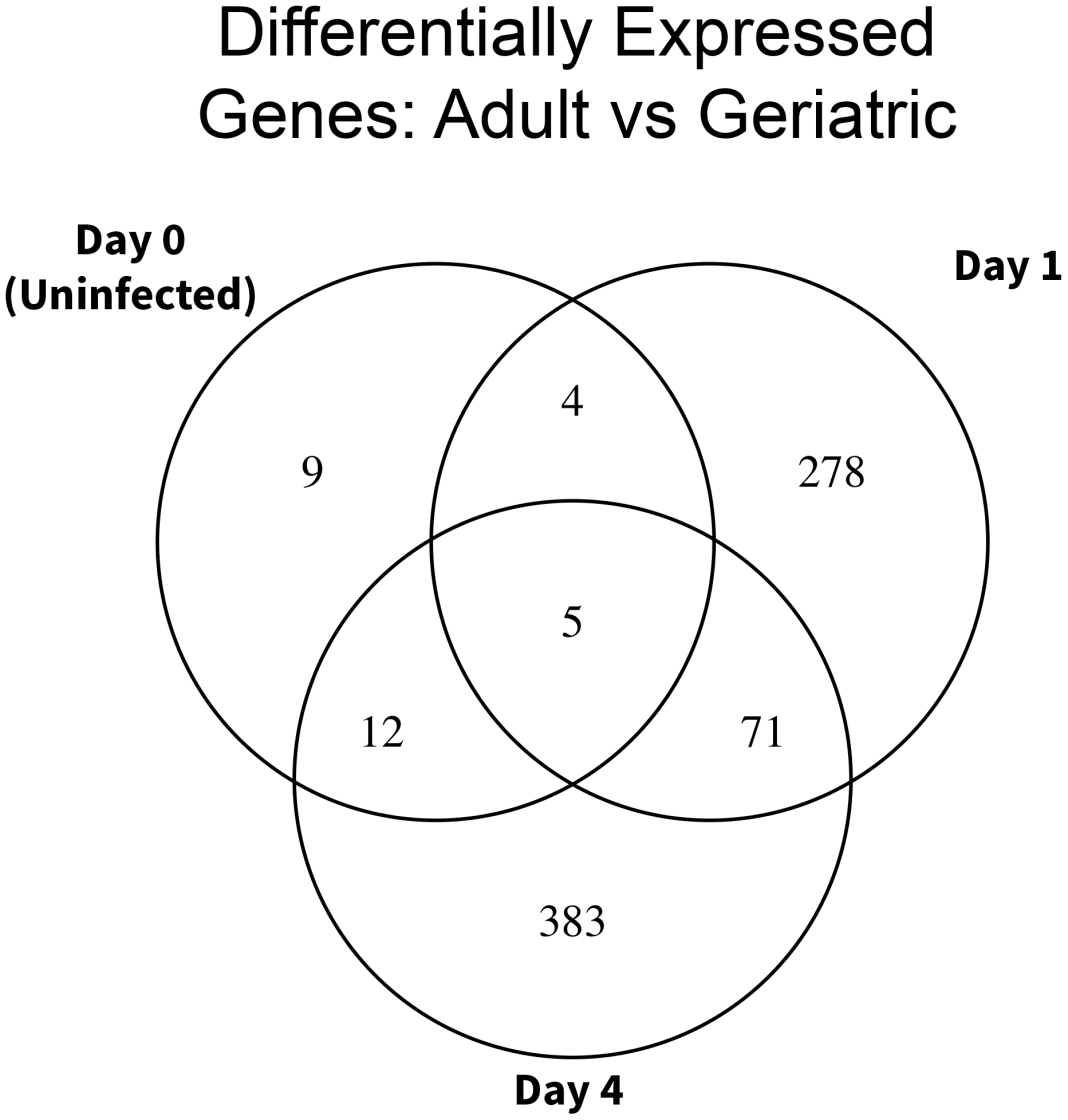
Numbers of differentially expressed genes between adult and geriatric cotton rats with FC > 2 or <0.5 and q-values of <0.05. Gene expression was compared between geriatric and adult cotton rats at day 0 (PBS treated), day 1, and day 4 post-RSV infection.

Lung transcriptional profiles were compared between age groups to identify potential immunological differences in geriatric cotton rats that could contribute to delayed RSV clearance. Gene expression was compared between geriatric and adult cotton rats at day 0 (PBS treated), day 1, and day 4 post-RSV infection, with DEGs defined as transcripts with a FC > 2 or <0.5 and *q*-values of <0.05 (Fig 2). Lungs of uninfected cotton rats demonstrated similar mRNA transcription between age groups, with only 30 DEGs. The proportion of DEGs between adult and geriatric cotton rats dramatically increased during RSV infection. At day 1 post-infection, 358 DEGs were identified, with 195 genes more highly expressed in geriatric cotton rats and 163 genes more highly expressed in adults. This Fig increased by day 4, when 471 DEGs were identified; 269 genes more highly expressed in geriatrics and 202 more highly expressed in adults (DEGs listed as S3–S5 Tables).

**Fig 3:**
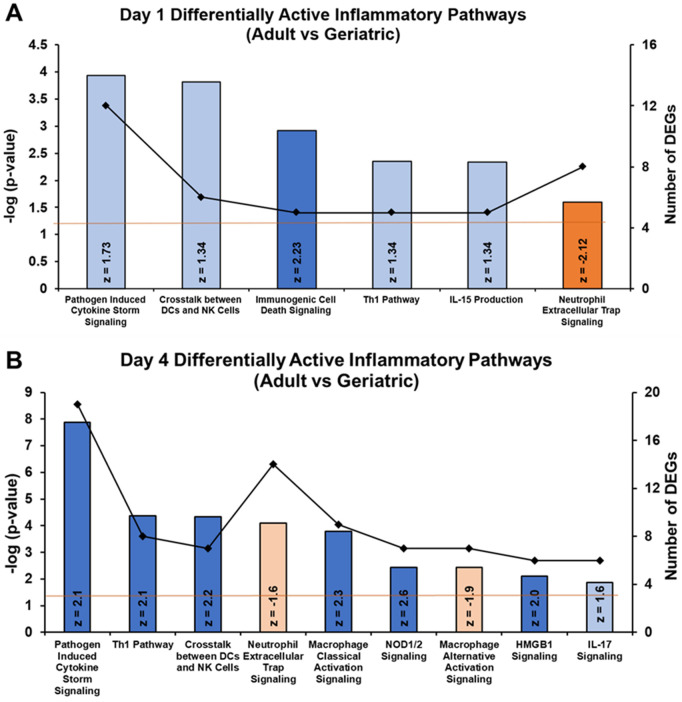
Differentially activated inflammatory pathways between adult and geriatric cotton rats at days 1 and 4 post-RSV infection. Pathway analysis was based on DEGs (*q* < 0.05, FC > 2 or <0.5) between the lung of adult and geriatric cotton rats at days 1 (A) or 4 (B) post-infection with RSV. The orange line represents the threshold for significance in pathway activity (*p* = 0.05, -log(*p*) = 1.3). Inflammatory pathways with significant differential expression and |z| > 1 are displayed (see S1–S2 Tables for top 50 differentially expressed pathways). Bars in dark blue are more active in adults (*z* > 2) while those in light blue trend higher in adults but are not directionally significant (0 < *z* < 2). Bars in dark orange are more active in geriatric cotton rats (*z* < -2) while those in light orange trend higher in geriatric cotton rats but are not directionally significant (0 > *z* > -2). Line with black diamonds (secondary y axis) represents the number of DEGs for each pathway. (*n* = 6 for all groups).

DEGs were used for pathway analysis, which demonstrated significantly lower enrichment of Pathogen Immunogenic Cell Death Signaling (Fig 3A), with several other inflammatory pathways differing significantly and trending higher in adult animals but without directional significance. The sole inflammatory pathway that was differentially enriched in geriatric cotton rats at day 1 was Neutrophil Extracellular Trap (NET) Signaling. Similarly, at day 4 post-infection, an expanded number of inflammatory and antiviral pathways were more highly enriched in adults compared to geriatric cotton rats (Fig 3B). Neutrophil Extracellular Trap Signaling and Macrophage Alternative Activation Signaling differed significantly between age groups and were more enriched in geriatrics to a degree that approached but did not reach directional significance. Collectively, these results show that RSV-infected geriatric cotton rats exhibit a lower inflammatory transcriptional profile compared to adults, including decreased enrichment of many of the pathways that are most active in RSV infection compared to baseline (Fig 1).

### Digital PCR of select inflammatory factors in adult and geriatric cotton rats

To corroborate mRNA-seq quantification results of key inflammatory factors, we performed ddPCR on RNA extracted from whole lung on days 1, 4, and 6 post-RSV infection, or day 1 post-inoculation with PBS (uninfected) measuring *CXCL10* (encoding IP-10), *CXCL1* (GRO), *IFNG* (IFN-γ), and *IL12A* (IL-12p35). IP-10 is well-characterized as a highly upregulated chemokine in RSV infection that plays an important role in generation of CD8 + T cell responses and RSV clearance [58–60,64,65]. Growth-related oncogene (GRO) is another chemokine that has been shown to be upregulated during RSV infection, with one study demonstrating increased expression in lungs of aged cotton rats at day 6 post-infection compared to adults [35,66,67]. IFN-γ plays critical roles as a generator and effector of Th1 function during viral infections, and its production has been shown to be lower in RSV-stimulated peripheral blood mononuclear cells of elderly humans [45]. A previous study in RSV-infected cotton rat lungs showed IFN-γ transcripts peaking earlier in infection in adults than geriatric animals, suggesting an age-associated delay in IFN-γ production [35]. IL-12 induces Th1 differentiation of CD4 + T cells and enhances CD8 + T cell activity and IFN-γ production during RSV infection [68]. IL-12p35 encodes the IL-12-specific alpha subunit, while IL-12B is a common subunit of both IL-12 and IL-23; as such, IL-12p35 was measured as a more specific marker of IL-12 expression.

**Fig 4:**
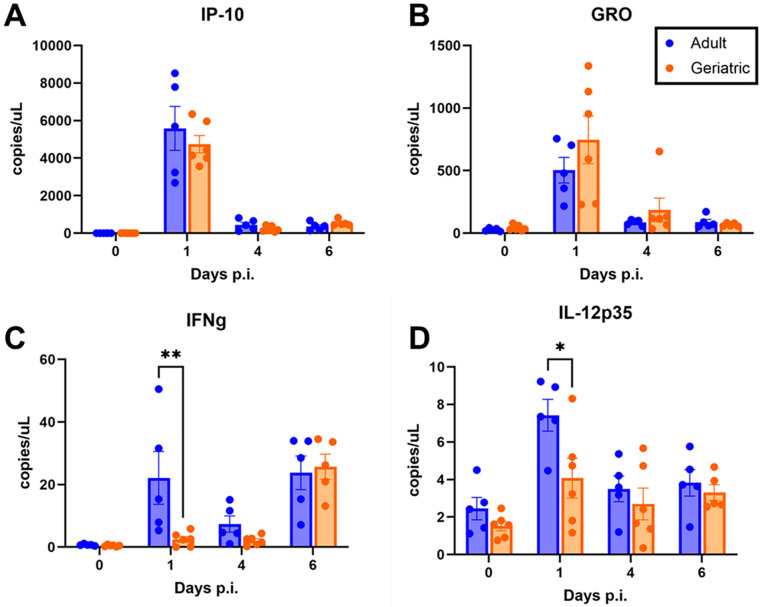
Digital PCR demonstrates lower expression of transcripts for Th1-associated cytokines early in RSV infection in geriatric cotton rats. Lung samples from adult or geriatric cotton rats at the indicated timepoints post-RSV infection were collected. Absolute quantification of gene expression for IP-10 (*CXCL10*), GRO (*CXCL1*), IFN-γ (*IFNG*), and IL-12p35 (*IL12A*) was performed by ddPCR of 300ng of cDNA for each sample. Chemokines IP-10 and GRO were upregulated early in RSV infection in both adult and geriatric cotton rats, with no age-associated differences. Th1 effector cytokine IFN-γ was upregulated at day 1 in adult but not geriatric cotton rats, with both age groups demonstrating an increase at day 6, around the time of viral clearance. Expression of IL-12p35 was also significantly lower in geriatric cotton rats at day 1 compared to adults. (*n* = 5 for all adult timepoints and geriatric day 6. *n* = 6 for geriatric days 0, 1, and 4).

**Fig 5:**
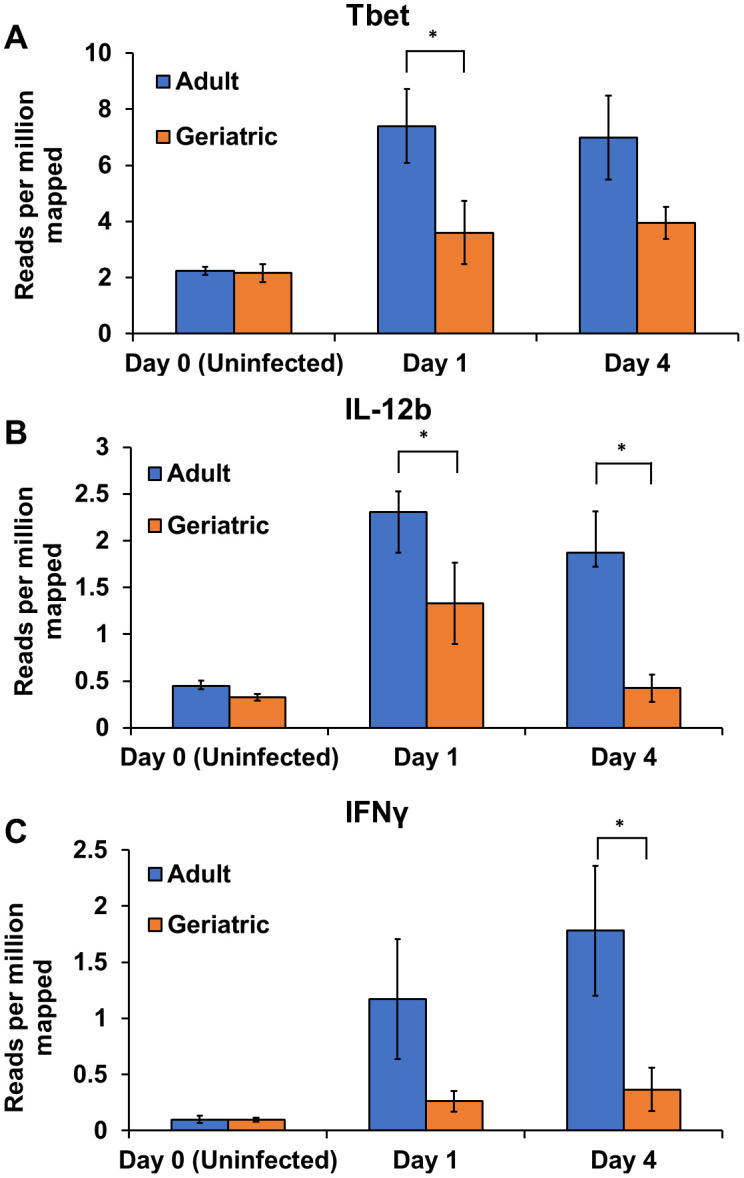
Reduced activation of the Th1 pathway in geriatric cotton rats. Expression of genes encoding Tbet (*TBX21*), IL-12b (*IL12B*), and IFN-γ (*IFNG*) in whole lung samples of adult or geriatric cotton rats at the indicated timepoints post-RSV infection are shown from RNA-seq libraries. Displayed reads per million mapped values are normalized using the trimmed mean of M values [56]. Expression of transcription factor *TBX21* was significantly lower in geriatric than adult cotton rats at day 1. *IL12B* was lower in geriatric cotton rats on both days 1 and 4 post-infection. *IFNG* expression was lower in geriatric cotton rats on day 4 (*q* = 0.1 on day 1). (*n* = 6 for all groups).

Transcripts for all cytokines were increased by day 1 post-RSV infection in adult lungs, with the most striking increase occurring in IP-10 (Fig 4A) as was observed by RNA-seq. IP-10 and GRO expression in adult and geriatric lungs were similar throughout RSV infection (Fig 4A-4B), but IFN-γ and IL-12p35 transcripts were significantly reduced in geriatric cotton rats at day 1 compared to adults (Fig 4C-4D). GRO and IP-10 expression measured by ddPCR and RNA-seq align in the lack of observed significant difference. IFN-γ expression was lower in geriatric cotton rats on day 1 post-infection by both ddPCR and RNA-seq (Fig 5), although RNA-seq also showed differential expression on day 4. RNA-seq did not show significantly different IL-12p35 on day 1, although a similar fold-change difference between age groups was observed (1.6-fold higher in adults, p = 0.2). Although IL-12p35 expression did not differ significantly by RNA-seq, expression of the IL-12 beta subunit was significantly lower in geriatric animals at days 1 and 4 post-infection (Fig 5). No age-associated differences in expression of transcripts for IP-10, GRO, IFN-γ, or IL-12p35 were observed on day 6 post-infection.

### RSV infection elicits reduced Th1 response in geriatric cotton rats

Generation of an effective Th1 response is critical for cell-mediated immunity and RSV clearance [69–72]. Pathway analysis of mRNA-seq data demonstrated a significant difference in the Th1 pathway between adult and geriatric cotton rats at days 1 and 4 post-RSV infection (Fig 3) based on five differentially expressed Th1-associated genes on day 1 and eight differentially expressed Th1-associated genes on day 4. Th1 pathway activity trended higher in adults on both days but was only directionally significant on day 4. Among the genes with increased upregulation in adult cotton rats compared to geriatrics were *TBX21* (encoding the transcription factor Tbet), *IL12B* encoding the beta chain of Th1 stimulating factor IL-12, and *IFNG* encoding the canonical effector cytokine IFN-γ (Fig 5). No age-associated differences in expression of these genes were observed in uninfected animals, but mean expression of all three factors was lower in geriatric cotton rats at days 1 and/or 4 post-infection. Decreased expression was significant at day 1 post-infection for *TBX21*, on both days 1 and 4 post-infection for *IL12B*, and day 4 post-infection for *IFNG*. *IL12B*, although associated with the Th1 pathway, is a common subunit of both IL-12 and IL-23 and is thus not a specific marker of IL-12 expression. No significant differences in expression of the alpha subunits of IL-12 or IL-23 were observed between age groups. The measured magnitude of elevated expression of *IFNG* in adults compared to geriatrics following RSV infection was similar by both qPCR (Fig 4) and RNA-seq, although there was greater variance within groups by qPCR resulting in lack of statistical significance.

### RSV-stimulated splenocytes of geriatric cotton rats show decreased IFN-γ production and increased IL-4 production

To assess Th1 and Th2 differentiation of CD4 + lymphocytes following RSV infection, we measured secretion of canonical Th1 and Th2 cytokines IFN-γ and IL-4 by stimulated splenocytes. Spleens were collected from adult and geriatric (>12 months of age) cotton rats at day 28 post-infection. Splenocytes were stimulated with UV-inactivated RSV antigen, with four replicates per animal. The supernatant of replicates was pooled, and IFN-γ and IL-4 were measured by ELISA. Stimulated splenocytes of all adult animals produced detectable levels of IFN-γ, while none produced detectable IL-4 (Fig 5). By contrast, only one of the three geriatric cotton rats produced detectable IFN-γ while all produced detectable IL-4 levels. This experiment was also performed on 5 younger geriatric cotton rats (>9 months, < 10 months of age) which did not result in production of detectable IL-4 upon stimulation in any individuals in the cohort. These results reinforce the results by RNA-seq demonstrating reduced Th1 activation during RSV infection in geriatric cotton rats (Figs 3B, 4). Further, IL-4 production upon stimulation indicates a Th2 response in geriatric cotton rats that was not observed in adults.

## Discussion

A number of mechanisms have been shown to contribute to prolonged and more severe RSV infections in elderly individuals, and geriatric cotton rats have been a useful tool for demonstrating altered immune parameters that mirror age-associated changes in humans. Geriatric cotton rats exhibit delayed RSV clearance [35–37], delayed CD8 + T cell responses [38], decreased neutralizing antibody titers and B cell responses [37,42], a delayed peak expression of genes encoding select cytokines including IFN-γ, IL-6, and IL-10 [35]. This study provides the first transcriptomic evaluation of expression profiles in the lung of RSV-infected adult and geriatric cotton rats. While the transcriptomic profiles of uninfected adult and geriatric animals are very similar, we find striking differences in expression of numerous inflammatory molecules and pathways during RSV infection between age groups. These age-associated differences are broadly characterized by a decrease in activity of pro-inflammatory pathways in geriatric cotton rats. On day 1 post-infection, pathway analysis of DEGs between the two age groups shows greater enrichment of Immunogenic Cell Death Signaling pathways in adults compared to geriatrics (Fig 3A). The list of differentially enriched pathways expands on day 4 post infection, with the Th1, Crosstalk between DCs and NK cells, Macrophage Classical Activation Signaling, NOD1/2 Signaling, and HMGB1 Signaling pathways all significantly more active in adults than geriatrics (Fig 3B).

**Fig 6:**
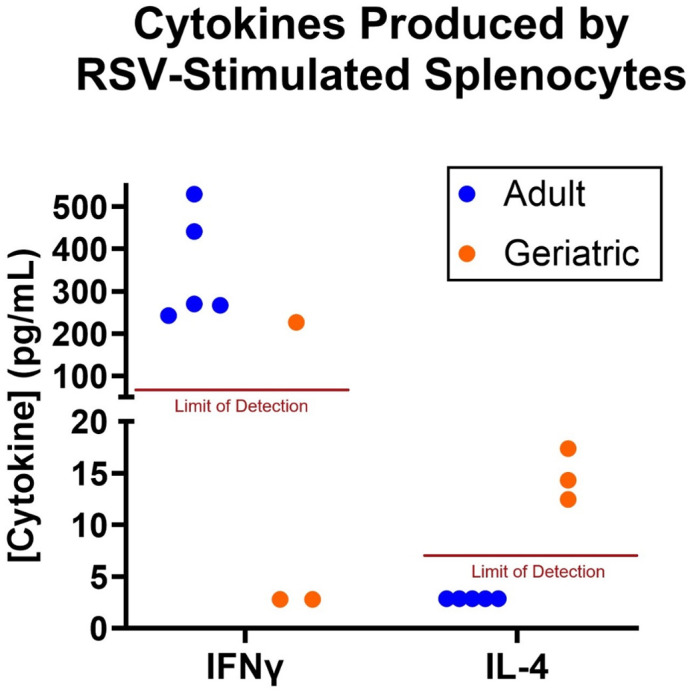
IFN-γ and IL-4 secretion by splenocytes of RSV-infected cotton rats. Spleens of adult and geriatric (>12 months of age for this cohort) cotton rats were collected at 28 days post-RSV infection. Following stimulation with UV-inactivated RSV antigen, splenocytes of adult cotton rats produced IFN-γ but no detectable IL-4. RSV antigen-stimulated splenocytes of geriatric cotton rats produced IL-4, but stimulation only induced detectable secretion of IFN-γ for 1 animal. (*n* = 5 for adult cotton rats, *n* = 3 for geriatric cotton rats).

One of the most consistent and potentially consequential findings was a reduction in expression of Th1-associated signaling molecules and cytokines in the geriatric age group (Fig 5). As is true for respiratory viruses in general, initiation of an effective Th1 response is critical for generation of cell-mediated immunity. IFN-γ promotes numerous protective effects during viral infections, including paracrine activation of immune cells, modulating differentiation and maturation of T cells, inducing production of antiviral molecules, and inhibiting virus release by infected host cells [73–79]. A delayed or reduced Th1 response would be predicted to impair the antiviral immune response both through reduced IFN-γ and CD8 + T cell responses, the major driver of RSV clearance [50]. In other at-risk groups including infants, impaired Th1 responses and IFN-γ production are correlated with worse clinical outcomes [80,81]. These more severe RSV infections stem not only from the aforementioned impairments to viral clearance, but also from a propensity to develop a dysregulated Th2-type immune response [82–84]. In addition to our data showing suppressed Th1 responses characterized by reduced expression of genes encoding Tbet, IL-12, and IFN-γ in geriatric cotton rats, we found that splenocytes of previously infected geriatric cotton rats demonstrated a Th2-skewed cytokine profile upon stimulation with RSV peptides (Fig 6). While Th2 predisposition is typically associated with hypersensitivity responses, eosinophilia, and mucus hypersecretion in infants, clinical consequences in the elderly are more likely characterized by immunosuppression and delayed viral clearance [82]. The Th2 pathway does not show significantly increased activation in geriatric cotton rat lungs at our measured timepoints compared to adults, but IL-4 production by stimulated splenocytes is characteristic of a Th2 response. We propose that a reduced Th1 response is the underlying consequential factor in skewing leukocyte differentiation.

One age-associated factor that paradoxically contributes to impaired immune responses in some conditions in elderly populations is a chronic state of low-level, sterile inflammation. This phenomenon is known as “inflammaging”, which is characterized by elevated pro-inflammatory markers in serum, including IL-1, IL-6, and TNF-α [85,86]. This dysregulated chronic inflammatory state in the elderly can impair immune responses to pathogens by increasing expression of inhibitory ligands such as PDL-1 on antigen presenting cells [87], recruiting and increasing the immunosuppressive function of Foxp3 + Treg cells [88–90], and decreasing T cell receptor signaling and histiocyte phagocytosis [91,92]. Some studies in lungs of old mice have shown evidence of chronic, age-associated inflammation through PCR and transcriptomic profiling [93,94]. Interestingly, the latter study found increased antiviral IFN pathway activation in RSV-infected old mice compared to young animals, contrasting with our observation in cotton rats. That study found a limited number of differentially expressed genes in infected animals overall, possibly reflecting the lower permissiveness and immune response to RSV infection in immunocompetent mice. Treatment of geriatric cotton rats with non-steroidal anti-inflammatory drugs alleviates the delayed clearance kinetics of RSV in geriatric cotton rats, initially suggesting that inflammaging could be a contributing factor to reduced immunity in our model [37]. However, this phenomenon appears to be tied to a specific pathway involving generation of elevated prostaglandin D_2_ in infected geriatric cotton rats with subsequent delay in antigen presentation and CD8 + T cell responses [38]. Our transcriptomic results in this study indicate that geriatric cotton rats, both uninfected and at the measured timepoints post-RSV infection, do not exhibit elevated inflammatory markers compared to adults. This finding is in line with previous studies demonstrating no difference in cellular inflammation between old and young cotton rats by lung histology and bronchoalveolar lavage cytology [37]. As such, although geriatric cotton rats do develop visceral fat inflammation and other metabolic disturbances such as insulin resistance [95], gene expression analysis does not show a broad age-associated increase in inflammation in the lungs of geriatric cotton rats.

Our results in cotton rats may be useful for identifying alterations in transcriptional profiles that contribute to impaired immune responses in the elderly. We demonstrate a reduced Th1 response in geriatric cotton rats infected with RSV, and a broadly reduced inflammatory response overall, compared to adults. This lower Th1 response may contribute to previously demonstrated deficiencies in geriatric cotton rats, including delayed CD8 + T cell responses and RSV clearance. Our model differs from natural RSV infections in elderly humans, who have been infected many times throughout life. Impaired responses to RSV in humans likely reflect deficiencies in secondary immune responses, whereas our model only assesses primary infections in cotton rats. Future studies in humans or more long-lived animal models are needed to assess the impact of alterations in activity of inflammatory pathways among the elderly on memory responses to RSV and other respiratory viruses.

## Declaration of competing interest

The authors have no known competing financial or personal interests to disclose. This work was supported by a Dean’s Distinguished graduate school fellowship (J.L.M) and research funds from the College of Veterinary Medicine at The Ohio State University (SN). The funders had no role in study design, data collection and analysis, decision to publish, or preparation of the manuscript.

## Supporting information

S1 Table Top 50 differentially expressed pathways between adult and geriatric cotton rats at day 1 post-RSV infection. (DOCX)

S2 Table Top 50 differentially expressed pathways between adult and geriatric cotton rats at day 4 post-RSV infection. (DOCX)

S3 Table Differentially expressed genes between adult and geriatric cotton rats at day 0 (uninfected) with FC > 2 or <0.5 and q-values of <0.05. (DOCX)

S4 Table Differentially expressed genes between adult and geriatric cotton rats at day 1 post-RSV infection with FC > 2 or <0.5 and q-values of <0.05. (DOCX)

S5 Table Differentially expressed genes between adult and geriatric cotton rats at day 4 post-RSV infection with FC > 2 or <0.5 and q-values of <0.05. (DOCX)
