# Supplementary material for: Reduced inflammatory and Th1 transcriptional profiles in geriatric versus adult cotton rats infected with respiratory syncytial virus
Source: PLoS Pathog. 2026 Jul 9;22(7):e1014323. doi: 10.1371/journal.ppat.1014323 (PMC13349118; doi:10.1371/journal.ppat.1014323)
Supplement: S1 Table — (DOCX) [file ppat.1014323.s001.docx]

| **Pathway** | **-log(p-value)** | **z-score** |
| --- | --- | --- |
| GP6 Signaling Pathway | 5.72 | 1.414 |
| S100 Family Signaling Pathway | 4.27 | -0.229 |
| Synaptogenesis Signaling Pathway | 3.94 | -1.265 |
| Pathogen Induced Cytokine Storm Signaling Pathway | 3.94 | 1.732 |
| Crosstalk between Dendritic Cells and Natural Killer Cells | 3.82 | 1.342 |
| CREB Signaling in Neurons | 3.49 | -0.535 |
| Hepatic Fibrosis / Hepatic Stellate Cell Activation | 3.48 | N/A |
| G-Protein Coupled Receptor Signaling | 3.29 | -0.775 |
| Pulmonary Fibrosis Idiopathic Signaling Pathway | 3.19 | 1 |
| Breast Cancer Regulation by Stathmin1 | 3.09 | -0.277 |
| Histidine Degradation VI | 2.98 | N/A |
| Immunogenic Cell Death Signaling Pathway | 2.92 | 2.236 |
| Phagosome Formation | 2.89 | -0.258 |
| Apelin Liver Signaling Pathway | 2.78 | N/A |
| Primary Immunodeficiency Signaling | 2.72 | N/A |
| Human Embryonic Stem Cell Pluripotency | 2.69 | -0.378 |
| FAK Signaling | 2.67 | 0.229 |
| Neuroprotective Role of THOP1 in Alzheimer's Disease | 2.36 | 1 |
| Th1 Pathway | 2.35 | 1.342 |
| IL-15 Production | 2.33 | 1.342 |
| Sertoli Cell-Sertoli Cell Junction Signaling | 2.24 | -1.134 |
| Role of Osteoblasts in Rheumatoid Arthritis Signaling Pathway | 2.23 | 0.378 |
| Intrinsic Prothrombin Activation Pathway | 2.23 | N/A |
| Role of Osteoclasts in Rheumatoid Arthritis Signaling Pathway | 2.22 | 2.121 |
| Gα12/13 Signaling | 2.19 | -0.447 |
| Macrophage Alternative Activation Signaling Pathway | 2.18 | 0 |
| Wound Healing Signaling Pathway | 2.15 | 1.89 |
| SNARE Signaling Pathway | 2.15 | -0.447 |
| Th2 Pathway | 2.14 | -0.447 |
| Granzyme B Signaling | 2.07 | N/A |
| CDX Gastrointestinal Cancer Signaling Pathway | 2.04 | 1.633 |
| Signaling by Rho Family GTPases | 2.02 | -1.134 |
| Microautophagy Signaling Pathway | 1.87 | N/A |
| MSP-RON Signaling Pathway | 1.84 | N/A |
| HOTAIR Regulatory Pathway | 1.83 | -0.447 |
| Pregnenolone Biosynthesis | 1.8 | N/A |
| β-alanine Degradation I | 1.76 | N/A |
| Threonine Degradation II | 1.76 | N/A |
| IL-12 Signaling and Production in Macrophages | 1.75 | 1.633 |
| Th1 and Th2 Activation Pathway | 1.74 | N/A |
| Tumoricidal Function of Hepatic Natural Killer Cells | 1.73 | N/A |
| Airway Pathology in Chronic Obstructive Pulmonary Disease | 1.69 | N/A |
| Ubiquinol-10 Biosynthesis (Eukaryotic) | 1.66 | N/A |
| Neutrophil Extracellular Trap Signaling Pathway | 1.6 | -2.121 |
| 4-aminobutyrate Degradation I | 1.58 | N/A |
| TR/RXR Activation | 1.58 | -2 |
| Role of Macrophages, Fibroblasts and Endothelial Cells in Rheumatoid Arthritis | 1.56 | N/A |
| Natural Killer Cell Signaling | 1.51 | -0.447 |
| Role of WNT/GSK-3β Signaling in the Pathogenesis of Influenza | 1.5 | N/A |
| Neurotrophin/TRK Signaling | 1.5 | N/A |

**Supplemental Table 1. Top 50 differentially expressed pathways between adult and geriatric cotton rats at day 1 post-RSV infection.** Pathway analysis is based on DEGs (*p* < 0.05, FC >2 or <0.5). Positive z scores reflect higher activity in adults, with z > |2| reflecting directional significance.
