# Supplementary material for: Reduced inflammatory and Th1 transcriptional profiles in geriatric versus adult cotton rats infected with respiratory syncytial virus
Source: PLoS Pathog. 2026 Jul 9;22(7):e1014323. doi: 10.1371/journal.ppat.1014323 (PMC13349118; doi:10.1371/journal.ppat.1014323)
Supplement: S2 Table — (DOCX) [file ppat.1014323.s002.docx]

| **Pathway** | **-log(p-value)** | **z-score** |
| --- | --- | --- |
| Granulocyte Adhesion and Diapedesis | 7.93 | N/A |
| Pathogen Induced Cytokine Storm Signaling Pathway | 7.88 | 2.065 |
| Agranulocyte Adhesion and Diapedesis | 7.35 | N/A |
| Hepatic Fibrosis / Hepatic Stellate Cell Activation | 6.89 | N/A |
| Phagosome Formation | 5.42 | -0.426 |
| FAK Signaling | 5.39 | -0.378 |
| Th1 and Th2 Activation Pathway | 4.88 | N/A |
| Th1 Pathway | 4.38 | 2.121 |
| Crosstalk between Dendritic Cells and Natural Killer Cells | 4.34 | 2.236 |
| Wound Healing Signaling Pathway | 4.16 | 1.508 |
| Neutrophil Extracellular Trap Signaling Pathway | 4.1 | -1.604 |
| Role of JAK1 and JAK3 in γc Cytokine Signaling | 4.09 | N/A |
| Glucocorticoid Receptor Signaling | 3.88 | N/A |
| G-Protein Coupled Receptor Signaling | 3.84 | -0.229 |
| Macrophage Classical Activation Signaling Pathway | 3.78 | 2.333 |
| S100 Family Signaling Pathway | 3.77 | -1.342 |
| Cardiac Hypertrophy Signaling (Enhanced) | 3.74 | 1.155 |
| Airway Pathology in Chronic Obstructive Pulmonary Disease | 3.63 | N/A |
| Role of Osteoblasts in Rheumatoid Arthritis Signaling Pathway | 3.61 | 0.632 |
| Role of hypercytokinemia/hyperchemokinemia in the Pathogenesis of Influenza | 3.56 | 2.449 |
| Role of Osteoclasts in Rheumatoid Arthritis Signaling Pathway | 3.4 | -0.302 |
| Breast Cancer Regulation by Stathmin1 | 3.3 | -1 |
| STAT3 Pathway | 3.28 | N/A |
| Multiple Sclerosis Signaling Pathway | 3.27 | 1.667 |
| Th2 Pathway | 3.24 | 1.134 |
| CREB Signaling in Neurons | 3.2 | -0.5 |
| cAMP-mediated signaling | 3.08 | -2.333 |
| PI3K/AKT Signaling | 2.93 | N/A |
| Role of Pattern Recognition Receptors in Recognition of Bacteria and Viruses | 2.91 | N/A |
| CDX Gastrointestinal Cancer Signaling Pathway | 2.9 | -2.121 |
| Activin Inhibin Signaling Pathway | 2.7 | -1.414 |
| GP6 Signaling Pathway | 2.67 | 0 |
| Role of Cytokines in Mediating Communication between Immune Cells | 2.63 | N/A |
| ABRA Signaling Pathway | 2.57 | -1.342 |
| Macrophage Alternative Activation Signaling Pathway | 2.44 | -1.89 |
| NOD1/2 Signaling Pathway | 2.44 | 2.646 |
| Pathogenesis of Multiple Sclerosis | 2.44 | N/A |
| Airway Inflammation in Asthma | 2.32 | N/A |
| Cellular Effects of Sildenafil (Viagra) | 2.32 | N/A |
| Semaphorin Neuronal Repulsive Signaling Pathway | 2.32 | -1.342 |
| Mitotic Roles of Polo-Like Kinase | 2.29 | 1 |
| PD-1, PD-L1 cancer immunotherapy pathway | 2.29 | 0 |
| Kinetochore Metaphase Signaling Pathway | 2.24 | 1 |
| Interferon Signaling | 2.22 | N/A |
| Pulmonary Fibrosis Idiopathic Signaling Pathway | 2.14 | -0.378 |
| HMGB1 Signaling | 2.1 | 2 |
| RHOGDI Signaling | 2.08 | 1 |
| VDR/RXR Activation | 2.05 | N/A |
| Maturity Onset Diabetes of Young (MODY) Signaling | 2.04 | N/A |
| Role of MAPK Signaling in Inhibiting the Pathogenesis of Influenza | 2.04 | 2 |

**Supplemental Table 2. Top 50 differentially expressed pathways between adult and geriatric cotton rats at day 4 post-RSV infection.** Pathway analysis is based on DEGs (*p* < 0.05, FC >2 or <0.5). Positive z scores reflect higher activity in adults, with z > |2| reflecting directional significance.
