# Supplementary material for: Reduced inflammatory and Th1 transcriptional profiles in geriatric versus adult cotton rats infected with respiratory syncytial virus
Source: PLoS Pathog. 2026 Jul 9;22(7):e1014323. doi: 10.1371/journal.ppat.1014323 (PMC13349118; doi:10.1371/journal.ppat.1014323)
Supplement: S3 Table — (DOCX) [file ppat.1014323.s003.docx]

| **GeneID** | **ENSID** | **GeneName** | **FC** | **FDR** |
| --- | --- | --- | --- | --- |
| Hispid2B021607 | ENSMUSP00000084656.4 | Kcnip4 | 2.479681 | 0.026224313 |
| Hispid2B025707 |  |  | 2.252905 | 0.033038192 |
| Hispid2B017674 |  |  | 2.180248 | 0.042188605 |
| Hispid2B022424 | ENSMUSP00000023356.6 | Snai2 | 2.08004 | 0.030135778 |
| Hispid2B000767 | ENSMUSP00000034868.7 | Clk1 | 0.492045 | 0.001107086 |
| Hispid2B024785 | ENSMUSP00000064823.7 | Klf2 | 0.487433 | 0.003096261 |
| Hispid2B007676 |  |  | 0.475389 | 0.012991771 |
| Hispid2B003468 | ENSMUSP00000033054.8 | Adm | 0.4747 | 0.024386983 |
| Hispid2B019155 | ENSMUSP00000040342.3 | Pcsk1n | 0.46731 | 0.030135778 |
| Hispid2B009672 | ENSMUSP00000005620.8 | Dnajb1 | 0.461722 | 0.018317984 |
| Hispid2B026862 | ENSMUSP00000035976.1 | Vax2 | 0.461604 | 0.016759521 |
| Hispid2B010659 |  |  | 0.449063 | 0.000352305 |
| Hispid2B021085 | ENSMUSP00000132366.1 | Apold1 | 0.417249 | 0.003096261 |
| Hispid2B013110 | ENSMUSP00000028166.2 | Nr4a2 | 0.38444 | 0.003112288 |
| Hispid2B007071 | ENSMUSP00000103245.2 | Klf4 | 0.374634 | 0.001471224 |
| Hispid2B024960 | ENSMUSP00000149214.1 | Csrnp1 | 0.373845 | 0.001471224 |
| Hispid2B028934 | ENSMUSP00000027941.8 | Atf3 | 0.360499 | 0.003096261 |
| Hispid2B030438 |  |  | 0.342566 | 0.000292012 |
| Hispid2B010193 | ENSMUSP00000076130.5 | Plk3 | 0.319914 | 0.000440258 |
| Hispid2B016442 | ENSMUSP00000034453.4 | Acta1 | 0.3112 | 0.004224013 |
| Hispid2B030393 | ENSMUSP00000044903.8 | Thbs1 | 0.290562 | 0.010534395 |
| Hispid2B000167 |  |  | 0.266108 | 0.024386983 |
| Hispid2ncA037570 |  |  | 0.248178 | 0.045213451 |
| Hispid2ncA039521 |  |  | 0.246472 | 0.003096261 |
| Hispid2B022469 | ENSMUSP00000123476.1 | Gm4846 | 0.233533 | 0.033037391 |
| Hispid2B024429 | ENSMUSP00000121455.1 | Nr4a3 | 0.167792 | 0.046300087 |
| Hispid2B002272 | ENSMUSP00000023779.6 | Nr4a1 | 0.155477 | 0.000352305 |
| Hispid2B005319 | ENSMUSP00000003640.2 | Fosb | 0.139208 | 0.035948045 |
| Hispid2B013327 | ENSMUSP00000153491.1 | Egr3 | 0.116966 | 0.014813872 |
| Hispid2B013705 | ENSMUSP00000027151.5 | Myl1 | 0.074331 | 0.000240053 |

**Supplemental Table 3. Differentially expressed genes between adult and geriatric cotton rats at day 0 (uninfected) with FC >2 or <0.5 and q-values of <0.05.** Green cells represent genes with higher expression in adults. Peach cells represent genes with higher expression in geriatrics.
