# Supplementary material for: Reduced inflammatory and Th1 transcriptional profiles in geriatric versus adult cotton rats infected with respiratory syncytial virus
Source: PLoS Pathog. 2026 Jul 9;22(7):e1014323. doi: 10.1371/journal.ppat.1014323 (PMC13349118; doi:10.1371/journal.ppat.1014323)
Supplement: S4 Table — (DOCX) [file ppat.1014323.s004.docx]

| **GeneID** | **ENSID** | **GeneName** | **FC** | **FDR** |
| --- | --- | --- | --- | --- |
| Hispid2B011042 | ENSMUSP00000035029.2 | Rbp2 | 9.131861185 | 0.017027959 |
| Hispid2B017856 |  |  | 8.491210608 | 0.001698177 |
| Hispid2B004901 |  |  | 6.589060033 | 0.004077184 |
| Hispid2B006941 | ENSMUSP00000101508.1 | Zfp683 | 6.225963672 | 0.013960971 |
| Hispid2B014914 | ENSMUSP00000137837.1 | Crtam | 5.351441977 | 0.028115346 |
| Hispid2B025309 | ENSMUSP00000070131.7 | Cd8b1 | 5.269260491 | 0.00213109 |
| Hispid2ncA039669 |  |  | 5.235003932 | 0.04115789 |
| Hispid2B028110 | ENSMUSP00000154022.1 | Cdh9 | 5.190426493 | 0.00010877 |
| Hispid2B009353 | ENSMUSP00000027860.7 | Xcl1 | 5.091751435 | 0.028718456 |
| Hispid2B015757 |  |  | 4.871004873 | 0.017804754 |
| Hispid2B025856 |  |  | 4.785582459 | 0.038279808 |
| Hispid2B011939 | ENSMUSP00000026159.5 | Cd7 | 4.570342671 | 0.01925406 |
| Hispid2B010445 | ENSMUSP00000037000.8 | Mcph1 | 4.52538041 | 0.016597684 |
| Hispid2ncA039208 |  |  | 4.397233205 | 0.049255175 |
| Hispid2B014139 | ENSMUSP00000064839.7 | Ttk | 4.277607932 | 0.035032637 |
| Hispid2B006778 | ENSMUSP00000005678.4 | Fcer2a | 4.238201019 | 0.002364802 |
| Hispid2B016512 |  |  | 4.217872823 | 0.048742396 |
| Hispid2B017764 |  |  | 4.170049964 | 0.011584948 |
| Hispid2B030531 | ENSMUSP00000041427.8 | Lefty1 | 4.134788353 | 0.000548153 |
| Hispid2B016841 | ENSMUSP00000045196.9 | Muc3 | 4.121038549 | 0.047360188 |
| Hispid2B027847 | ENSMUSP00000022213.7 | Thbs4 | 4.074203623 | 0.01983161 |
| Hispid2B019900 | ENSMUSP00000034602.7 | Cd3d | 4.009248337 | 0.013135755 |
| Hispid2B021888 |  |  | 4.008727153 | 0.016455846 |
| Hispid2B005255 |  |  | 3.897578277 | 0.02044655 |
| Hispid2B003159 |  |  | 3.884843834 | 5.31E-05 |
| Hispid2ncA040071 |  |  | 3.86547646 | 0.030570762 |
| Hispid2B031466 | ENSMUSP00000130632.2 | Siglech | 3.855642009 | 0.018896792 |
| Hispid2B023557 | ENSMUSP00000112472.1 | Col6a4 | 3.813208295 | 0.009594908 |
| Hispid2B007620 | ENSMUSP00000125867.1 | Il12b | 3.771906038 | 0.04115789 |
| Hispid2B016161 | ENSMUSP00000117263.1 | Lrrtm4 | 3.737078798 | 0.015695344 |
| Hispid2B016609 |  |  | 3.719755078 | 0.045016666 |
| Hispid2B017051 | ENSMUSP00000058535.4 | Serpina9 | 3.691830186 | 0.021770772 |
| Hispid2B012430 | ENSMUSP00000110523.1 | Pacsin1 | 3.654550681 | 0.029680934 |
| Hispid2B025629 | ENSMUSP00000045637.2 | Rims4 | 3.558273535 | 0.025063338 |
| Hispid2B013592 | ENSMUSP00000082952.5 | Lefty2 | 3.544642181 | 0.03263808 |
| Hispid2B008005 |  |  | 3.51651667 | 0.035185233 |
| Hispid2B015782 |  |  | 3.512915607 | 0.041080564 |
| Hispid2B018882 | ENSMUSP00000036731.7 | Atf7ip2 | 3.509041628 | 0.049216332 |
| Hispid2B015491 | ENSMUSP00000043957.7 | Lama1 | 3.489796176 | 0.019881671 |
| Hispid2B002655 | ENSMUSP00000127024.1 | Ccr9 | 3.430515676 | 0.019991062 |
| Hispid2B028540 | ENSMUSP00000039376.6 | Exo1 | 3.400146544 | 0.020464311 |
| Hispid2B006354 |  |  | 3.369834936 | 0.002972476 |
| Hispid2B006601 |  |  | 3.313478263 | 0.017228747 |
| Hispid2B017444 | ENSMUSP00000127292.1 | Cdhr5 | 3.313001105 | 0.034914111 |
| Hispid2B006114 | ENSMUSP00000115127.2 | Sycp2l | 3.269852529 | 0.031213232 |
| Hispid2B013223 | ENSMUSP00000021793.7 | Elovl2 | 3.257932645 | 0.03880579 |
| Hispid2B011673 | ENSMUSP00000020920.3 | Rgs9 | 3.228064014 | 0.042653101 |
| Hispid2B010664 | ENSMUSP00000039088.8 | Vsx1 | 3.188861902 | 0.013963242 |
| Hispid2B024504 | ENSMUSP00000001484.2 | Tbx21 | 3.146210398 | 0.030990295 |
| Hispid2B006538 |  |  | 3.136374336 | 0.024926984 |
| Hispid2B019604 | ENSMUSP00000027933.5 | Dtl | 3.131952469 | 0.008755891 |
| Hispid2ncA039869 |  |  | 3.093675726 | 0.04468954 |
| Hispid2B027985 |  |  | 3.082562278 | 0.014614225 |
| Hispid2B012469 | ENSMUSP00000053751.7 | Sprr1a | 3.066202804 | 5.31E-05 |
| Hispid2B013773 |  |  | 3.027418057 | 0.048676067 |
| Hispid2B013890 |  |  | 3.019920567 | 0.011663175 |
| Hispid2B007972 | ENSMUSP00000112808.1 | Gpr174 | 3.007239677 | 0.007146773 |
| Hispid2B027363 | ENSMUSP00000137492.1 | Tmem95 | 2.981521059 | 0.040231459 |
| Hispid2B004619 | ENSMUSP00000108662.2 | Uhrf1 | 2.963414493 | 0.01052343 |
| Hispid2ncA039006 |  |  | 2.949612444 | 0.037953824 |
| Hispid2B029423 | ENSMUSP00000108804.2 | Afm | 2.942024977 | 0.021727047 |
| Hispid2B014310 |  |  | 2.934108087 | 0.04223489 |
| Hispid2B020996 |  |  | 2.931145778 | 0.020923863 |
| Hispid2B015409 |  |  | 2.912337209 | 0.018231792 |
| Hispid2B020053 |  |  | 2.886640885 | 0.037335107 |
| Hispid2B009440 | ENSMUSP00000037466.7 | Cd160 | 2.872266864 | 0.015812989 |
| Hispid2B011926 |  |  | 2.872101209 | 0.028492008 |
| Hispid2B018567 | ENSMUSP00000020040.3 | Nts | 2.865236588 | 0.049886842 |
| Hispid2B020132 | ENSMUSP00000056720.3 | Ifnb1 | 2.85233279 | 0.000724841 |
| Hispid2B019569 | ENSMUSP00000001547.7 | Col1a1 | 2.85054868 | 6.98E-05 |
| Hispid2B022116 |  |  | 2.828573536 | 0.042285584 |
| Hispid2B001697 | ENSMUSP00000033310.7 | Mki67 | 2.806411157 | 0.030324551 |
| Hispid2B014761 | ENSMUSP00000099443.4 | Skap1 | 2.804187457 | 0.000389029 |
| Hispid2B002091 |  |  | 2.803018135 | 0.04196376 |
| Hispid2B030090 | ENSMUSP00000027291.4 | Zap70 | 2.778358359 | 0.048386049 |
| Hispid2B015868 | ENSMUSP00000088196.4 | Syt6 | 2.771756165 | 0.014817138 |
| Hispid2B024889 |  |  | 2.749733482 | 0.006480727 |
| Hispid2B005545 |  |  | 2.748280576 | 0.021460674 |
| Hispid2B025735 |  |  | 2.746908514 | 0.047979085 |
| Hispid2B000737 | ENSMUSP00000125069.1 | Col22a1 | 2.726504172 | 0.02496163 |
| Hispid2B027945 | ENSMUSP00000143005.1 | Cspg5 | 2.722277158 | 0.010983518 |
| Hispid2B003706 | ENSMUSP00000099896.1 | Cd3e | 2.71976661 | 0.013113963 |
| Hispid2B031332 |  |  | 2.705823476 | 0.046804258 |
| Hispid2B030611 | ENSMUSP00000099423.3 | Ccr7 | 2.702835511 | 0.00808001 |
| Hispid2B013276 | ENSMUSP00000043896.7 | Chtf18 | 2.699925965 | 0.009859815 |
| Hispid2B008643 | ENSMUSP00000047164.7 | Capn8 | 2.698973643 | 0.019255532 |
| Hispid2B025891 | ENSMUSP00000060129.4 | Themis | 2.668201054 | 0.016205195 |
| Hispid2B002025 | ENSMUSP00000029456.4 | Cd2 | 2.653367521 | 0.016205195 |
| Hispid2B017674 |  |  | 2.652753726 | 0.001961184 |
| Hispid2B005925 |  |  | 2.649422515 | 0.015353985 |
| Hispid2B029609 | ENSMUSP00000026661.3 | Tk1 | 2.625908571 | 0.020867558 |
| Hispid2B031282 | ENSMUSP00000020350.8 | Lgr5 | 2.587657542 | 0.00072348 |
| Hispid2B027644 | ENSMUSP00000009396.6 | Tspan32 | 2.58632726 | 0.014431782 |
| Hispid2B026563 | ENSMUSP00000024118.4 | Clec4n | 2.584943357 | 0.016772833 |
| Hispid2B024746 | ENSMUSP00000072660.4 | Sctr | 2.582093877 | 0.049886842 |
| Hispid2B027195 |  |  | 2.535000689 | 0.040459115 |
| Hispid2B015385 |  |  | 2.505548007 | 0.045721299 |
| Hispid2B028130 | ENSMUSP00000015581.4 | Gzmb | 2.504061148 | 0.021770772 |
| Hispid2B026175 | ENSMUSP00000037477.7 | Myrfl | 2.487744197 | 0.014716537 |
| Hispid2B004427 |  |  | 2.484219197 | 0.047867482 |
| Hispid2B030934 | ENSMUSP00000153311.1 | Gas1 | 2.477697859 | 0.047596745 |
| Hispid2B028045 | ENSMUSP00000034625.5 | Chek1 | 2.473048205 | 0.003568355 |
| Hispid2B013802 | ENSMUSP00000038877.6 | Pclaf | 2.460325666 | 0.027696893 |
| Hispid2B012438 | ENSMUSP00000074988.3 | Cenph | 2.457516356 | 0.027434444 |
| Hispid2B000146 | ENSMUSP00000005255.2 | Ccn4 | 2.440465947 | 0.021834808 |
| Hispid2B016162 | ENSMUSP00000023897.5 | Gzma | 2.419115087 | 0.02179044 |
| Hispid2B023376 |  |  | 2.418344148 | 0.047867482 |
| Hispid2B006836 | ENSMUSP00000099752.4 | Cdca7 | 2.386245476 | 0.000698823 |
| Hispid2B007181 |  |  | 2.379089375 | 0.047555735 |
| Hispid2B002336 | ENSMUSP00000046012.7 | Apln | 2.363979832 | 0.014093091 |
| Hispid2B030893 | ENSMUSP00000037045.2 | Sstr1 | 2.36113842 | 0.025875709 |
| Hispid2B021403 | ENSMUSP00000097648.2 | Samd5 | 2.357098894 | 0.019053218 |
| Hispid2B024999 | ENSMUSP00000041483.5 | Prf1 | 2.355944157 | 0.016930499 |
| Hispid2B009197 | ENSMUSP00000029440.8 | Olfml3 | 2.347469353 | 0.002083193 |
| Hispid2B013410 | ENSMUSP00000048383.3 | Kif4 | 2.339410038 | 0.042421066 |
| Hispid2B030749 | ENSMUSP00000125693.2 | C6 | 2.328181651 | 0.025331393 |
| Hispid2B015546 | ENSMUSP00000047284.8 | Tespa1 | 2.322689459 | 0.008872771 |
| Hispid2B025952 | ENSMUSP00000009390.3 | Trpm5 | 2.311900811 | 0.007651095 |
| Hispid2B028631 |  |  | 2.30322449 | 0.003937872 |
| Hispid2B005742 | ENSMUSP00000091469.2 | Cdc6 | 2.286699595 | 0.019295875 |
| Hispid2B015016 | ENSMUSP00000055930.5 | Hs3st3a1 | 2.27342504 | 0.019426452 |
| Hispid2B023560 |  |  | 2.268694235 | 0.03802848 |
| Hispid2B012452 | ENSMUSP00000126422.1 | Ms4a1 | 2.257873648 | 0.00348246 |
| Hispid2B010373 | ENSMUSP00000048239.4 | Cd79b | 2.256481008 | 0.001999233 |
| Hispid2B019536 |  |  | 2.255464903 | 0.049984271 |
| Hispid2B011409 |  |  | 2.255210783 | 0.014898953 |
| Hispid2B002344 | ENSMUSP00000110652.3 | Xpnpep2 | 2.252512171 | 0.003747256 |
| Hispid2B025206 |  |  | 2.24587592 | 0.013653168 |
| Hispid2B017548 | ENSMUSP00000031668.8 | Col1a2 | 2.240452149 | 0.001120949 |
| Hispid2B017590 |  |  | 2.232008867 | 0.000475649 |
| Hispid2B017029 | ENSMUSP00000032207.8 | Klrg1 | 2.221866674 | 0.035888619 |
| Hispid2B022715 | ENSMUSP00000066822.7 | Penk | 2.221766283 | 0.003778592 |
| Hispid2B029451 | ENSMUSP00000031674.7 | Tfpi2 | 2.220246459 | 0.01925406 |
| Hispid2B023144 | ENSMUSP00000000028.7 | Cdc45 | 2.219735203 | 0.043606351 |
| Hispid2B002201 | ENSMUSP00000031668.8 | Col1a2 | 2.218423541 | 8.29E-05 |
| Hispid2B007189 | ENSMUSP00000058021.4 | Timeless | 2.208068405 | 0.023918115 |
| Hispid2B018793 | ENSMUSP00000131480.1 | Kcnf1 | 2.207022219 | 0.032033269 |
| Hispid2B030678 |  |  | 2.201058532 | 0.030946134 |
| Hispid2B013781 | ENSMUSP00000145803.1 | Cd19 | 2.196245507 | 0.021461861 |
| Hispid2B013579 | ENSMUSP00000094951.4 | Msh5 | 2.189531975 | 0.013003112 |
| Hispid2B031545 | ENSMUSP00000101921.1 | Septin1 | 2.152475999 | 0.030545647 |
| Hispid2B013791 |  |  | 2.146275136 | 0.025889467 |
| Hispid2B008280 | ENSMUSP00000020549.2 | Gzmm | 2.140819408 | 0.02309955 |
| Hispid2B030846 | ENSMUSP00000071627.5 | Frem1 | 2.135392194 | 0.010983518 |
| Hispid2B010177 | ENSMUSP00000123336.1 | Hal | 2.132185809 | 0.012364732 |
| Hispid2B015166 | ENSMUSP00000047845.4 | Dctpp1 | 2.13196122 | 0.018542033 |
| Hispid2B019782 | ENSMUSP00000079222.5 | Zfp551 | 2.126418438 | 0.03094132 |
| Hispid2B007681 |  |  | 2.126015003 | 0.013480676 |
| Hispid2B024934 | ENSMUSP00000100933.2 | Nr1h4 | 2.124778952 | 0.021367923 |
| Hispid2B006736 | ENSMUSP00000001147.4 | Col6a1 | 2.105716709 | 0.000129382 |
| Hispid2B024170 |  |  | 2.101418171 | 0.015695344 |
| Hispid2B019239 | ENSMUSP00000028663.4 | Creb3l1 | 2.101283113 | 0.000247959 |
| Hispid2B026181 | ENSMUSP00000126826.1 | Scn2b | 2.086177382 | 0.039063144 |
| Hispid2ncA038832 |  |  | 2.070157694 | 0.048008263 |
| Hispid2B020793 | ENSMUSP00000106809.1 | Efr3b | 2.069530908 | 0.000332089 |
| Hispid2B028918 | ENSMUSP00000045344.8 | Clspn | 2.059988829 | 0.011928198 |
| Hispid2B024696 | ENSMUSP00000046028.7 | Tmc3 | 2.05900125 | 0.013003112 |
| Hispid2B031812 | ENSMUSP00000086112.3 | Olfm4 | 2.051730548 | 0.036622931 |
| Hispid2B019820 | ENSMUSP00000028897.7 | Cpxm1 | 2.037232964 | 0.027817587 |
| Hispid2B015239 | ENSMUSP00000047894.6 | Inhba | 2.035744858 | 0.048227968 |
| Hispid2B001738 |  |  | 2.014662239 | 0.016851803 |
| Hispid2B013552 | ENSMUSP00000104860.2 | Itk | 2.003020604 | 0.04964094 |
| Hispid2B022293 | ENSMUSP00000021918.8 | Ror2 | 2.002674174 | 0.000175981 |
| Hispid2B008787 | ENSMUSP00000105275.2 | Fam227a | 0.49754718 | 0.042681435 |
| Hispid2B024275 | ENSMUSP00000005769.6 | Tmod4 | 0.497195465 | 0.046860895 |
| Hispid2B017057 |  |  | 0.496611422 | 0.023438131 |
| Hispid2B029374 | ENSMUSP00000119447.1 | Slc26a2 | 0.496557775 | 0.01525656 |
| Hispid2B010687 |  |  | 0.495725203 | 0.001731459 |
| Hispid2B027514 | ENSMUSP00000108225.1 | Dpp10 | 0.491462607 | 0.040709926 |
| Hispid2B008507 | ENSMUSP00000019931.5 | Lrp11 | 0.491094666 | 0.006151022 |
| Hispid2B004412 | ENSMUSP00000109523.1 | Gspt2 | 0.48955236 | 0.043102871 |
| Hispid2B019573 |  |  | 0.486830773 | 0.027665977 |
| Hispid2B018376 | ENSMUSP00000040405.3 | Vwa1 | 0.485130332 | 0.016601268 |
| Hispid2B019237 | ENSMUSP00000122846.1 | Arl9 | 0.48479206 | 0.003570546 |
| Hispid2B001482 | ENSMUSP00000070322.5 | Syt5 | 0.484734817 | 0.044472152 |
| Hispid2B008444 | ENSMUSP00000059026.6 | Six1 | 0.482049721 | 0.026411204 |
| Hispid2B009734 | ENSMUSP00000101718.1 | Bnip3 | 0.481531998 | 0.046391079 |
| Hispid2B020070 | ENSMUSP00000006431.6 | Atp6v1b1 | 0.480261463 | 0.017831034 |
| Hispid2B020457 | ENSMUSP00000097318.3 | Lgals12 | 0.479087322 | 0.022223114 |
| Hispid2B005591 | ENSMUSP00000023083.7 | Cyp2d22 | 0.479031793 | 0.016601268 |
| Hispid2B023198 |  |  | 0.476679637 | 0.020600633 |
| Hispid2B012419 | ENSMUSP00000032386.4 | Bhlhe41 | 0.47350088 | 0.003526487 |
| Hispid2B011644 | ENSMUSP00000021685.6 | Hhipl1 | 0.47310488 | 0.016436568 |
| Hispid2B008017 | ENSMUSP00000058354.9 | Msx1 | 0.472616671 | 0.014093091 |
| Hispid2B021178 | ENSMUSP00000006716.6 | Wnt6 | 0.471133125 | 0.01188323 |
| Hispid2B016451 |  |  | 0.471050086 | 0.017131401 |
| Hispid2B000944 | ENSMUSP00000020586.6 | Slc22a4 | 0.470626891 | 0.000191543 |
| Hispid2B006488 | ENSMUSP00000064246.6 | Chst1 | 0.470108314 | 0.045721299 |
| Hispid2B015171 | ENSMUSP00000097060.3 | Duox1 | 0.467242619 | 0.00135577 |
| Hispid2B028715 | ENSMUSP00000048555.7 | Cpe | 0.466240676 | 0.000558976 |
| Hispid2B025265 | ENSMUSP00000136572.1 | Adssl1 | 0.465955672 | 0.022958108 |
| Hispid2B010334 |  |  | 0.465285163 | 0.008367994 |
| Hispid2B014129 |  |  | 0.464940717 | 0.048008263 |
| Hispid2B003515 | ENSMUSP00000123025.2 | Exoc3l2 | 0.460885964 | 0.038044964 |
| Hispid2B001090 | ENSMUSP00000087511.4 | Sybu | 0.460684344 | 0.000475649 |
| Hispid2B031137 | ENSMUSP00000097219.3 | Ano3 | 0.460306041 | 0.04115789 |
| Hispid2ncA040057 |  |  | 0.457878903 | 0.038552866 |
| Hispid2B028235 | ENSMUSP00000042857.3 | Dnah3 | 0.457177975 | 0.049255175 |
| Hispid2B006780 | ENSMUSP00000009538.5 | Syn2 | 0.455680013 | 0.000318581 |
| Hispid2B007466 | ENSMUSP00000083242.6 | Scarb1 | 0.452184034 | 0.001494891 |
| Hispid2B008273 | ENSMUSP00000128828.1 | Cdkn1c | 0.451835 | 0.013474689 |
| Hispid2B006586 | ENSMUSP00000048829.8 | Cdh26 | 0.45131917 | 0.030528914 |
| Hispid2B002492 | ENSMUSP00000113680.1 | Fam189a1 | 0.447728401 | 0.044427772 |
| Hispid2B001204 | ENSMUSP00000063548.7 | Abat | 0.44231821 | 0.023229853 |
| Hispid2B029883 | ENSMUSP00000110440.1 | Fkbp5 | 0.442147843 | 0.018480315 |
| Hispid2B003083 | ENSMUSP00000027997.3 | Rgs5 | 0.440557691 | 0.032012466 |
| Hispid2B016907 |  |  | 0.437827257 | 0.000902762 |
| Hispid2B025332 | ENSMUSP00000029377.7 | Tm4sf4 | 0.435771497 | 0.005017312 |
| Hispid2B015901 | ENSMUSP00000011178.2 | Slc5a1 | 0.435539634 | 0.01983161 |
| Hispid2ncA037080 |  |  | 0.434815093 | 0.00335481 |
| Hispid2ncA038101 |  |  | 0.433655928 | 0.003846766 |
| Hispid2B006677 | ENSMUSP00000036227.5 | St6galnac5 | 0.430981348 | 0.043124314 |
| Hispid2B016745 |  |  | 0.4289232 | 0.002782981 |
| Hispid2B013110 | ENSMUSP00000028166.2 | Nr4a2 | 0.428558161 | 0.01976174 |
| Hispid2B003577 |  |  | 0.427313973 | 0.046264291 |
| Hispid2B023829 | ENSMUSP00000060411.6 | Mrgprg | 0.424930022 | 0.048034544 |
| Hispid2B020231 | ENSMUSP00000028389.3 | Frzb | 0.418709275 | 0.00802813 |
| Hispid2B019343 |  |  | 0.41863294 | 0.027285278 |
| Hispid2B013483 |  |  | 0.417867026 | 0.039063144 |
| Hispid2B009583 | ENSMUSP00000145092.1 | Cyp26b1 | 0.417101507 | 0.019991062 |
| Hispid2B026514 |  |  | 0.416028204 | 0.024294428 |
| Hispid2B020616 |  |  | 0.412562814 | 0.009594908 |
| Hispid2B019571 | ENSMUSP00000129869.1 | Slc6a2 | 0.411081561 | 0.027434444 |
| Hispid2ncA036763 |  |  | 0.407711255 | 0.030770236 |
| Hispid2B004314 | ENSMUSP00000103222.2 | Srcin1 | 0.407135051 | 0.011448073 |
| Hispid2B028091 | ENSMUSP00000031707.7 | Aass | 0.406692225 | 0.010075073 |
| Hispid2B030438 |  |  | 0.404589908 | 8.29E-05 |
| Hispid2B012300 |  |  | 0.404498845 | 0.001031947 |
| Hispid2B007591 |  |  | 0.404219287 | 0.011668386 |
| Hispid2B026105 | ENSMUSP00000042716.8 | Nkx6-1 | 0.403311105 | 0.04092689 |
| Hispid2B022847 | ENSMUSP00000105833.2 | Esrrb | 0.401191877 | 0.003568355 |
| Hispid2B002086 |  |  | 0.398563151 | 0.009594908 |
| Hispid2B022433 | ENSMUSP00000029946.7 | Rragd | 0.398337878 | 0.000548153 |
| Hispid2B023332 | ENSMUSP00000045332.6 | Itprid1 | 0.398078088 | 0.016813753 |
| Hispid2B010631 |  |  | 0.397388739 | 0.010070469 |
| Hispid2B002857 | ENSMUSP00000066864.6 | Cdh22 | 0.396645277 | 0.032098369 |
| Hispid2ncA038430 |  |  | 0.395897006 | 0.048421159 |
| Hispid2B012329 | ENSMUSP00000026259.9 | Pitx3 | 0.395710329 | 0.02615397 |
| Hispid2B020496 | ENSMUSP00000022522.8 | Tdh | 0.395390996 | 0.027221153 |
| Hispid2B029142 | ENSMUSP00000081867.6 | Dnah11 | 0.395292338 | 0.039751227 |
| Hispid2B013246 | ENSMUSP00000032489.7 | Ltbr | 0.395001025 | 0.049364847 |
| Hispid2B022140 | ENSMUSP00000056990.6 | Ric3 | 0.39490454 | 0.014817138 |
| Hispid2B012186 | ENSMUSP00000105561.1 | Syndig1 | 0.391860542 | 0.009864685 |
| Hispid2B021880 | ENSMUSP00000123025.2 | Exoc3l2 | 0.391809998 | 0.047867482 |
| Hispid2B022040 |  |  | 0.387740558 | 0.000921969 |
| Hispid2B029091 |  |  | 0.385870877 | 0.009631804 |
| Hispid2B008409 |  |  | 0.384781362 | 0.021864507 |
| Hispid2B011604 | ENSMUSP00000043753.7 | Ttll7 | 0.377466874 | 0.000124783 |
| Hispid2ncA039139 |  |  | 0.376179633 | 0.042401779 |
| Hispid2B025241 | ENSMUSP00000000619.6 | Clcn4 | 0.366236983 | 0.000129382 |
| Hispid2ncA036942 |  |  | 0.365004375 | 0.043124314 |
| Hispid2B009742 | ENSMUSP00000132092.1 | Dpy19l2 | 0.362911914 | 0.023666436 |
| Hispid2B020280 | ENSMUSP00000059705.5 | Adra2c | 0.359945178 | 0.019236736 |
| Hispid2B022394 | ENSMUSP00000031215.8 | Brdt | 0.359180526 | 0.030639441 |
| Hispid2B017413 | ENSMUSP00000032185.7 | Slc6a6 | 0.358206564 | 0.005631622 |
| Hispid2ncA039621 |  |  | 0.356325231 | 0.010884306 |
| Hispid2B020537 | ENSMUSP00000112769.1 | Fam107a | 0.356168464 | 0.00116753 |
| Hispid2B018872 | ENSMUSP00000145700.1 | Fam71e1 | 0.355384739 | 0.007146773 |
| Hispid2B001800 | ENSMUSP00000056967.7 | Sall3 | 0.354057487 | 0.044491328 |
| Hispid2ncA040007 |  |  | 0.350474653 | 0.042580193 |
| Hispid2ncA037437 |  |  | 0.346095821 | 0.00072348 |
| Hispid2B028056 | ENSMUSP00000105149.1 | Vstm2l | 0.344659042 | 0.020456893 |
| Hispid2B025994 | ENSMUSP00000020679.2 | Nipal4 | 0.342976309 | 0.002203032 |
| Hispid2B008651 | ENSMUSP00000031131.9 | Uchl1 | 0.342088956 | 0.033554427 |
| Hispid2B011882 | ENSMUSP00000104132.2 | Hif3a | 0.339997182 | 0.039219541 |
| Hispid2B029175 | ENSMUSP00000032732.8 | Apba2 | 0.339678721 | 0.011705591 |
| Hispid2B016467 | ENSMUSP00000004505.2 | Npc1l1 | 0.337701354 | 0.005553308 |
| Hispid2B008032 | ENSMUSP00000032958.7 | Ucp3 | 0.337221378 | 0.001461051 |
| Hispid2B013157 |  |  | 0.33609174 | 0.028055152 |
| Hispid2B029038 | ENSMUSP00000035250.2 | Alox12b | 0.329437321 | 0.016397015 |
| Hispid2B015235 |  |  | 0.328656844 | 0.027434444 |
| Hispid2B017477 |  |  | 0.328585258 | 0.006480727 |
| Hispid2B018252 | ENSMUSP00000141553.1 | Tnr | 0.321238623 | 0.027230795 |
| Hispid2B028137 | ENSMUSP00000031535.4 | Hnf1a | 0.319962085 | 0.047915524 |
| Hispid2B003450 | ENSMUSP00000112630.1 | Hcrtr1 | 0.299657918 | 0.040709926 |
| Hispid2B020032 | ENSMUSP00000046191.7 | Elmod1 | 0.298758142 | 0.000700684 |
| Hispid2B026862 | ENSMUSP00000035976.1 | Vax2 | 0.298148093 | 0.00010344 |
| Hispid2B022677 | ENSMUSP00000021932.5 | Drd1 | 0.297877782 | 0.039063144 |
| Hispid2ncA039521 |  |  | 0.295954656 | 0.019236736 |
| Hispid2B028287 |  |  | 0.293751552 | 0.030718063 |
| Hispid2ncA040074 |  |  | 0.290194871 | 0.046860895 |
| Hispid2B011280 | ENSMUSP00000150887.1 | Zbtb16 | 0.287566401 | 0.002875936 |
| Hispid2ncA039580 |  |  | 0.281898029 | 0.048008263 |
| Hispid2B026924 | ENSMUSP00000119872.1 | Tmem63c | 0.275004333 | 0.029921752 |
| Hispid2B006251 | ENSMUSP00000141648.1 | Fgg | 0.267890962 | 0.033521066 |
| Hispid2B016442 | ENSMUSP00000034453.4 | Acta1 | 0.265873977 | 0.001417044 |
| Hispid2B027157 | ENSMUSP00000084958.2 | Hmx1 | 0.264684328 | 0.048676067 |
| Hispid2B017368 |  |  | 0.261167009 | 0.046663959 |
| Hispid2B029522 | ENSMUSP00000021691.4 | Degs2 | 0.260317497 | 0.003190373 |
| Hispid2B008829 | ENSMUSP00000128113.1 | Pglyrp4 | 0.257562337 | 0.033019062 |
| Hispid2B030506 |  |  | 0.256907346 | 0.002043685 |
| Hispid2B019155 | ENSMUSP00000040342.3 | Pcsk1n | 0.25143641 | 6.98E-05 |
| Hispid2B005454 | ENSMUSP00000109403.1 | Itgb2l | 0.242506342 | 0.01925406 |
| Hispid2B004866 |  |  | 0.24049968 | 0.036770275 |
| Hispid2B001058 |  |  | 0.240074062 | 0.031701497 |
| Hispid2B011811 |  |  | 0.239932113 | 0.049732662 |
| Hispid2ncA040122 |  |  | 0.239599244 | 0.000791395 |
| Hispid2B009735 |  |  | 0.235266097 | 0.00010877 |
| Hispid2B026069 |  |  | 0.231083163 | 0.013135755 |
| Hispid2ncA039186 |  |  | 0.224655545 | 0.026754178 |
| Hispid2B010797 | ENSMUSP00000140081.1 | Syt2 | 0.223988382 | 0.049786588 |
| Hispid2ncA037601 |  |  | 0.22284577 | 0.011996962 |
| Hispid2B022165 | ENSMUSP00000087805.4 | Muc15 | 0.222016762 | 0.026190846 |
| Hispid2ncA039683 |  |  | 0.217141916 | 0.009056466 |
| Hispid2B019688 |  |  | 0.216585492 | 0.04038842 |
| Hispid2B029439 |  |  | 0.215760428 | 0.001882053 |
| Hispid2ncA037993 |  |  | 0.214613118 | 0.013386774 |
| Hispid2ncA039587 |  |  | 0.212580075 | 0.018411264 |
| Hispid2B004325 | ENSMUSP00000015583.1 | Ctsg | 0.210606317 | 0.046564034 |
| Hispid2B027990 | ENSMUSP00000057488.2 | Dmrta1 | 0.210221184 | 0.049693805 |
| Hispid2ncA038796 |  |  | 0.209500772 | 0.004279116 |
| Hispid2B014114 | ENSMUSP00000127778.1 | Ttll6 | 0.200563719 | 0.020360998 |
| Hispid2B006311 | ENSMUSP00000044245.6 | Prdm14 | 0.192173201 | 0.03417506 |
| Hispid2B014965 |  |  | 0.18879533 | 0.011093469 |
| Hispid2ncA037081 |  |  | 0.185296707 | 0.000921969 |
| Hispid2B010049 |  |  | 0.18519074 | 0.04223489 |
| Hispid2B029107 |  |  | 0.183105608 | 0.027696893 |
| Hispid2B017501 | ENSMUSP00000106811.2 | Trim50 | 0.182029519 | 0.033732749 |
| Hispid2ncA038378 |  |  | 0.178190509 | 0.003568355 |
| Hispid2ncA037644 |  |  | 0.177976531 | 0.044846172 |
| Hispid2B029295 | ENSMUSP00000078757.5 | Ntrk2 | 0.171555789 | 0.020181564 |
| Hispid2B007778 | ENSMUSP00000080521.6 | Zdhhc12 | 0.166109671 | 0.021198344 |
| Hispid2B031166 | ENSMUSP00000135855.3 | Nptxr | 0.164888042 | 8.29E-05 |
| Hispid2B010637 |  |  | 0.158934531 | 0.017347309 |
| Hispid2ncA039471 |  |  | 0.154903485 | 0.001428265 |
| Hispid2ncA038328 |  |  | 0.15271168 | 0.02362634 |
| Hispid2B027681 |  |  | 0.146716349 | 0.04071319 |
| Hispid2ncA038918 |  |  | 0.144956339 | 0.008872771 |
| Hispid2B026628 | ENSMUSP00000107499.4 | Pik3c2g | 0.143354095 | 0.0023381 |
| Hispid2B005276 | ENSMUSP00000033189.3 | Cckbr | 0.14151957 | 0.008992834 |
| Hispid2ncA037733 |  |  | 0.141376195 | 0.027817587 |
| Hispid2B028323 | ENSMUSP00000063136.4 | Htr1f | 0.137697497 | 0.018542033 |
| Hispid2B021636 |  |  | 0.137621024 | 0.005286905 |
| Hispid2B011766 |  |  | 0.129941439 | 0.017831034 |
| Hispid2B016098 |  |  | 0.115574547 | 0.031755724 |
| Hispid2B031093 | ENSMUSP00000085794.5 | Pkhd1 | 0.111591956 | 0.001031947 |
| Hispid2B024445 | ENSMUSP00000007340.2 | Atp12a | 0.110428079 | 0.001999233 |
| Hispid2ncA039022 |  |  | 0.109922667 | 0.003937872 |
| Hispid2B017039 |  |  | 0.107781361 | 0.036996507 |
| Hispid2ncA036683 |  |  | 0.105604447 | 0.002780513 |
| Hispid2B017365 | ENSMUSP00000026548.7 | Adgra1 | 0.105445157 | 0.013135755 |
| Hispid2B016754 |  |  | 0.098797769 | 0.041080564 |
| Hispid2B027086 |  |  | 0.095707902 | 0.00962678 |
| Hispid2B029866 |  |  | 0.089378818 | 0.043961313 |
| Hispid2B026025 |  |  | 0.086498136 | 0.039063144 |
| Hispid2B002491 |  |  | 0.085472137 | 0.038381891 |
| Hispid2ncA036550 |  |  | 0.08286069 | 6.26E-05 |
| Hispid2ncA039849 |  |  | 0.082674401 | 0.000902762 |
| Hispid2ncA037347 |  |  | 0.077614387 | 0.015222094 |
| Hispid2ncA039613 |  |  | 0.072336323 | 0.000389029 |
| Hispid2B016709 |  |  | 0.066853465 | 0.005691284 |
| Hispid2ncA039690 |  |  | 0.061294221 | 0.000467948 |
| Hispid2ncA037570 |  |  | 0.058193069 | 9.76E-06 |
| Hispid2B007470 |  |  | 0.053953004 | 0.045974619 |
| Hispid2ncA035816 |  |  | 0.052301482 | 0.000373819 |
| Hispid2ncA038150 |  |  | 0.045439499 | 0.000332089 |
| Hispid2ncA038158 |  |  | 0.044028706 | 0.000597141 |
| Hispid2B013705 | ENSMUSP00000027151.5 | Myl1 | 0.035448639 | 3.28E-05 |

**Supplemental Table 4. Differentially expressed genes between adult and geriatric cotton rats at day 1 post-RSV infection with FC >2 or <0.5 and q-values of <0.05.** Green cells represent genes with higher expression in adults. Peach cells represent genes with higher expression in geriatrics.
