# Supplementary material for: Reduced inflammatory and Th1 transcriptional profiles in geriatric versus adult cotton rats infected with respiratory syncytial virus
Source: PLoS Pathog. 2026 Jul 9;22(7):e1014323. doi: 10.1371/journal.ppat.1014323 (PMC13349118; doi:10.1371/journal.ppat.1014323)
Supplement: S5 Table — (DOCX) [file ppat.1014323.s005.docx]

| **GeneID** | **ENSID** | **GeneName** | **FC** | **FDR** |
| --- | --- | --- | --- | --- |
| Hispid2B028547 | ENSMUSP00000061095.7 | Kdm5d | 216.6057965 | 0.041221614 |
| Hispid2B025781 | ENSMUSP00000088729.5 | Ddx3y | 179.1629263 | 0.047300627 |
| Hispid2B002603 | ENSMUSP00000070012.7 | Uty | 110.0324818 | 0.035653219 |
| Hispid2B006686 | ENSMUSP00000088717.1 | Sry | 38.72269063 | 0.037624142 |
| Hispid2ncA039669 |  |  | 18.05953967 | 0.023435935 |
| Hispid2ncA039435 |  |  | 10.24061825 | 0.013633552 |
| Hispid2B015242 |  |  | 9.540232015 | 0.002059153 |
| Hispid2ncA037874 |  |  | 9.024269276 | 0.030392246 |
| Hispid2B006787 |  |  | 7.123715792 | 0.024943463 |
| Hispid2ncA038679 |  |  | 7.026033271 | 0.005196663 |
| Hispid2B002091 |  |  | 6.934145356 | 0.00067019 |
| Hispid2B005806 | ENSMUSP00000029275.5 | Il2 | **6.80317308** | **0.001647015** |
| Hispid2B006126 |  |  | 5.997312956 | 0.0009726 |
| Hispid2B021371 | ENSMUSP00000063800.3 | Ifng | **5.867327915** | **0.028076715** |
| Hispid2B031809 |  |  | 5.750966364 | 0.00561768 |
| Hispid2B021103 | ENSMUSP00000131415.1 | Fabp7 | 5.669571649 | 0.032598096 |
| Hispid2B031466 | ENSMUSP00000130632.2 | Siglech | 5.609521097 | 0.006411162 |
| Hispid2B025551 |  |  | 5.278672022 | 0.006428264 |
| Hispid2B016675 | ENSMUSP00000017255.3 | Krt24 | 5.270211972 | 0.0089178 |
| Hispid2B021923 | ENSMUSP00000136529.1 | Ms4a18 | 5.262465602 | 0.033604962 |
| Hispid2ncA039147 |  |  | 5.207848697 | 0.002507438 |
| Hispid2B009497 | ENSMUSP00000020243.7 | Ascl1 | 5.096489117 | 0.021490298 |
| Hispid2B030617 | ENSMUSP00000027449.4 | Nppc | 4.956405958 | 0.02493378 |
| Hispid2B007620 | ENSMUSP00000125867.1 | Il12b | **4.94730542** | **0.017439299** |
| Hispid2ncA039065 |  |  | 4.94453745 | 0.0230499 |
| Hispid2ncA036819 |  |  | 4.887865181 | 0.016555986 |
| Hispid2B023911 | ENSMUSP00000058020.8 | Onecut1 | 4.849135867 | 0.003250733 |
| Hispid2B016476 | ENSMUSP00000103782.2 | Etv2 | 4.826606045 | 0.027355941 |
| Hispid2B000260 | ENSMUSP00000044272.6 | Septin14 | 4.709073349 | 0.015261672 |
| Hispid2B007531 | ENSMUSP00000125965.1 | Sectm1b | 4.683940667 | 0.017227404 |
| Hispid2B028874 | ENSMUSP00000055562.2 | Nccrp1 | 4.607332739 | 0.000113049 |
| Hispid2B025925 | ENSMUSP00000142520.1 | Fgfbp1 | 4.590076798 | 0.007916873 |
| Hispid2B021819 | ENSMUSP00000124251.1 | Mgat4f | 4.575730138 | 0.019410029 |
| Hispid2B010780 | ENSMUSP00000114904.1 | Aire | 4.360135311 | 0.032598096 |
| Hispid2B025629 | ENSMUSP00000045637.2 | Rims4 | 4.333437843 | 0.004189911 |
| Hispid2B015757 |  |  | 4.268603081 | 0.011183557 |
| Hispid2B028110 | ENSMUSP00000154022.1 | Cdh9 | 4.150847916 | 0.034189961 |
| Hispid2B014461 |  |  | 4.130786244 | 0.009445718 |
| Hispid2ncA039999 |  |  | 4.115865391 | 0.036281369 |
| Hispid2B016609 |  |  | 4.097733268 | 0.015269437 |
| Hispid2B031677 | ENSMUSP00000108716.3 | Cxcl9 | 4.07474684 | 0.015889517 |
| Hispid2ncA036292 |  |  | 4.067736283 | 0.017081955 |
| Hispid2B003171 | ENSMUSP00000048180.6 | Acd | 4.041544079 | 0.025652148 |
| Hispid2B024903 |  |  | 4.011526643 | 0.012266339 |
| Hispid2B019981 | ENSMUSP00000124668.1 | Il21 | 3.979543504 | 0.031964432 |
| Hispid2ncA029368 |  |  | 3.96713784 | 0.041221614 |
| Hispid2B013035 |  |  | 3.935481197 | 0.00628694 |
| Hispid2B000959 | ENSMUSP00000084196.4 | Gpr55 | 3.887166015 | 0.004515466 |
| Hispid2B018882 | ENSMUSP00000036731.7 | Atf7ip2 | 3.810876669 | 0.039493626 |
| Hispid2B019861 | ENSMUSP00000108446.1 | Il13ra2 | 3.777208798 | 0.020675352 |
| Hispid2B022084 | ENSMUSP00000086731.3 | Gpr15 | 3.753727528 | 0.031548083 |
| Hispid2B004182 | ENSMUSP00000038473.2 | Ccr8 | 3.685955584 | 0.02008298 |
| Hispid2B013081 | ENSMUSP00000111239.2 | Lix1 | 3.680553503 | 0.019036336 |
| Hispid2B019373 | ENSMUSP00000130949.1 | Otog | 3.661931997 | 0.027578399 |
| Hispid2B014262 | ENSMUSP00000127514.1 | Myh4 | 3.658527041 | 0.041085975 |
| Hispid2B017444 | ENSMUSP00000127292.1 | Cdhr5 | 3.589985357 | 0.048487577 |
| Hispid2B031296 | ENSMUSP00000025266.5 | Lta | 3.586133623 | 0.010063956 |
| Hispid2B019187 |  |  | 3.560741227 | 0.027355941 |
| Hispid2B027093 | ENSMUSP00000139868.1 | Lexm | 3.522738205 | 0.047003268 |
| Hispid2B029104 |  |  | 3.501643067 | 0.007778038 |
| Hispid2B004410 | ENSMUSP00000087102.3 | Astl | 3.500580623 | 0.021669725 |
| Hispid2B015754 | ENSMUSP00000022108.7 | Hapln1 | 3.456962211 | 0.045170264 |
| Hispid2B009353 | ENSMUSP00000027860.7 | Xcl1 | 3.368646583 | 0.025186863 |
| Hispid2B002461 |  |  | 3.337221698 | 0.010835019 |
| Hispid2B005067 | ENSMUSP00000047646.6 | Cxcl10 | 3.332536338 | 0.004244295 |
| Hispid2B006295 |  |  | 3.317091427 | 0.040781552 |
| Hispid2B015034 | ENSMUSP00000052444.6 | Cxcr3 | 3.309830416 | 0.00670022 |
| Hispid2B020686 | ENSMUSP00000039912.6 | Deup1 | 3.299163509 | 0.035491052 |
| Hispid2B011939 | ENSMUSP00000026159.5 | Cd7 | 3.295779162 | 0.01243014 |
| Hispid2ncA037526 |  |  | 3.294467809 | 0.049220389 |
| Hispid2B009518 | ENSMUSP00000052095.6 | Col26a1 | 3.294085645 | 0.012030853 |
| Hispid2B023784 |  |  | 3.292639391 | 0.004818533 |
| Hispid2B017183 |  |  | 3.26259161 | 0.014175338 |
| Hispid2B000823 | ENSMUSP00000023775.7 | Cela1 | 3.246120965 | 0.040248045 |
| Hispid2B000807 |  |  | 3.217157407 | 0.003258877 |
| Hispid2ncA040162 |  |  | 3.207832675 | 0.01659496 |
| Hispid2B003831 |  |  | 3.183452736 | 0.015626429 |
| Hispid2B009774 |  |  | 3.174217814 | 0.029292194 |
| Hispid2B001143 | ENSMUSP00000028111.4 | Il2ra | 3.141974813 | 0.006879585 |
| Hispid2B022801 | ENSMUSP00000040611.10 | Tcfl5 | 3.132633568 | 0.01243014 |
| Hispid2B022103 |  |  | 3.100650604 | 0.032996999 |
| Hispid2ncA040109 |  |  | 3.08958928 | 0.040450512 |
| Hispid2B000324 | ENSMUSP00000149084.1 | Igdcc3 | 3.064883628 | 0.000588408 |
| Hispid2B017296 |  |  | 3.050518312 | 0.004834606 |
| Hispid2B003548 |  |  | 3.048049891 | 0.039563284 |
| Hispid2B012881 | ENSMUSP00000106120.2 | Slc12a1 | 3.02141272 | 0.042470031 |
| Hispid2B022884 | ENSMUSP00000060463.6 | Shisal2a | 2.99355581 | 0.047632327 |
| Hispid2ncA037030 |  |  | 2.975168767 | 0.032350069 |
| Hispid2B016161 | ENSMUSP00000117263.1 | Lrrtm4 | 2.969774937 | 0.0089178 |
| Hispid2B011856 |  |  | 2.941814047 | 0.035589815 |
| Hispid2B014914 | ENSMUSP00000137837.1 | Crtam | 2.920475619 | 0.025243808 |
| Hispid2ncA038991 |  |  | 2.919930306 | 0.02757721 |
| Hispid2ncA039446 |  |  | 2.915403901 | 0.036334156 |
| Hispid2ncA038364 |  |  | 2.898432872 | 0.02493378 |
| Hispid2B015868 | ENSMUSP00000088196.4 | Syt6 | 2.894308624 | 0.001139221 |
| Hispid2B016418 | ENSMUSP00000028665.4 | Patl2 | 2.87032916 | 0.01073157 |
| Hispid2B031417 |  |  | 2.862970168 | 0.032631777 |
| Hispid2B012046 | ENSMUSP00000050669.3 | Zmym1 | 2.813220388 | 0.028076715 |
| Hispid2B009722 |  |  | 2.797453876 | 0.01026148 |
| Hispid2B000654 | ENSMUSP00000027690.6 | Avpr1b | 2.758396328 | 0.010034113 |
| Hispid2B023419 | ENSMUSP00000023068.6 | Smc1b | 2.739351929 | 0.015743059 |
| Hispid2B009440 | ENSMUSP00000037466.7 | Cd160 | 2.715841834 | 0.016694308 |
| Hispid2B019060 | ENSMUSP00000099605.3 | Il22ra1 | 2.715751486 | 0.030707664 |
| Hispid2B019536 |  |  | 2.714338527 | 0.004189911 |
| Hispid2ncA038613 |  |  | 2.711367637 | 0.021107377 |
| Hispid2B006449 | ENSMUSP00000024858.5 | Galnt14 | 2.707766288 | 0.003434767 |
| Hispid2B004928 | ENSMUSP00000085346.1 | Nms | 2.697635772 | 0.019727247 |
| Hispid2B009509 | ENSMUSP00000034408.5 | Gpr83 | 2.694673488 | 0.037783056 |
| Hispid2B031151 | ENSMUSP00000025844.4 | Ctsw | 2.674411094 | 0.008423021 |
| Hispid2B001979 | ENSMUSP00000038297.4 | Klhdc8a | 2.668538865 | 0.003093364 |
| Hispid2B012804 | ENSMUSP00000039376.6 | Exo1 | 2.661729109 | 0.033971706 |
| Hispid2B003329 | ENSMUSP00000021610.5 | Chga | 2.65961479 | 0.014248554 |
| Hispid2B002165 |  |  | 2.645015497 | 0.00359841 |
| Hispid2B029113 | ENSMUSP00000093770.4 | Tigit | 2.62840674 | 0.022722148 |
| Hispid2ncA036410 |  |  | 2.622147895 | 0.033067879 |
| Hispid2B014041 |  |  | 2.608918116 | 0.044737782 |
| Hispid2B018531 |  |  | 2.604249772 | 0.008322146 |
| Hispid2B002835 | ENSMUSP00000006476.4 | Upk1a | 2.572347059 | 0.013897553 |
| Hispid2B010841 |  |  | 2.564840171 | 0.02852677 |
| Hispid2B017856 |  |  | 2.556767952 | 0.01772163 |
| Hispid2ncA040052 |  |  | 2.505329399 | 0.029921227 |
| Hispid2B022116 |  |  | 2.498174534 | 0.025314265 |
| Hispid2B025309 | ENSMUSP00000070131.7 | Cd8b1 | 2.476007704 | 0.010789854 |
| Hispid2B007785 | ENSMUSP00000146932.1 | Erfl | 2.444352039 | 0.039789672 |
| Hispid2B019260 | ENSMUSP00000129467.2 | Otogl | 2.443012194 | 0.013572793 |
| Hispid2B017174 | ENSMUSP00000099360.3 | Gh | 2.440450881 | 0.02486386 |
| Hispid2B015493 |  |  | 2.432566478 | 0.012558734 |
| Hispid2B028753 |  |  | 2.419318581 | 0.020675352 |
| Hispid2B019841 | ENSMUSP00000058896.7 | Ddit4l | 2.415588045 | 0.030730114 |
| Hispid2B006941 | ENSMUSP00000101508.1 | Zfp683 | 2.407078001 | 0.037178869 |
| Hispid2B029831 |  |  | 2.406797615 | 0.022630352 |
| Hispid2B003172 | ENSMUSP00000082207.6 | Tlr9 | 2.394898449 | 0.047003268 |
| Hispid2B027454 | ENSMUSP00000032217.1 | Lag3 | 2.378692037 | 0.025953942 |
| Hispid2B006354 |  |  | 2.353672549 | 0.003250733 |
| Hispid2B018598 | ENSMUSP00000079358.7 | Rpp25 | 2.342928195 | 0.010155813 |
| Hispid2B028313 | ENSMUSP00000119185.1 | Il9r | 2.341809114 | 0.037144354 |
| Hispid2B013236 | ENSMUSP00000108235.1 | Galnt5 | 2.333254153 | 0.01013274 |
| Hispid2B023557 | ENSMUSP00000112472.1 | Col6a4 | 2.31694561 | 0.033089396 |
| Hispid2B004584 | ENSMUSP00000082548.4 | Isg15 | 2.312632721 | 0.020949349 |
| Hispid2B028739 | ENSMUSP00000039758.5 | B4galnt4 | 2.301259667 | 0.024799328 |
| Hispid2B011089 | ENSMUSP00000036797.8 | Olig2 | 2.29883264 | 0.02523463 |
| Hispid2B009951 |  |  | 2.29549718 | 0.017649064 |
| Hispid2B030611 | ENSMUSP00000099423.3 | Ccr7 | 2.274797044 | 0.029767993 |
| Hispid2B026917 | ENSMUSP00000029368.2 | Ccna1 | 2.274222464 | 0.044495314 |
| Hispid2B008728 | ENSMUSP00000046539.4 | Camk4 | 2.269183043 | 0.034269259 |
| Hispid2B006831 |  |  | 2.266042219 | 0.015330582 |
| Hispid2B013850 | ENSMUSP00000141891.1 | Kcnk2 | 2.261483662 | 0.011038115 |
| Hispid2ncA036586 |  |  | 2.258935103 | 0.021152422 |
| Hispid2B026740 | ENSMUSP00000097150.4 | Mis18a | 2.249719795 | 0.007778038 |
| Hispid2B015818 |  |  | 2.237822157 | 0.020507679 |
| Hispid2B002957 |  |  | 2.236685589 | 0.016373263 |
| Hispid2B005166 | ENSMUSP00000049725.5 | Cldn23 | 2.235895522 | 0.020949349 |
| Hispid2B017512 | ENSMUSP00000074201.4 | Brinp3 | 2.226921287 | 0.025755999 |
| Hispid2B020220 | ENSMUSP00000006565.6 | Cdc20 | 2.223262103 | 0.012673647 |
| Hispid2B009270 |  |  | 2.215264127 | 0.012077412 |
| Hispid2B001351 | ENSMUSP00000030372.5 | Col9a2 | 2.211486302 | 0.020382451 |
| Hispid2B028484 | ENSMUSP00000024270.7 | Cdca3 | 2.202983841 | 0.003847542 |
| Hispid2B000172 |  |  | 2.20084052 | 0.020196716 |
| Hispid2B019218 |  |  | 2.198510284 | 0.039775135 |
| Hispid2B017621 |  |  | 2.18339116 | 0.031337051 |
| Hispid2B025707 |  |  | 2.181296142 | 0.020196716 |
| Hispid2B020053 |  |  | 2.180428248 | 0.029540843 |
| Hispid2B001139 | ENSMUSP00000105140.1 | Bcl11a | 2.172022003 | 0.03963574 |
| Hispid2B016871 | ENSMUSP00000113216.1 | Depdc1a | 2.167300035 | 0.022487652 |
| Hispid2B000794 |  |  | 2.138907154 | 0.006879585 |
| Hispid2ncA038494 |  |  | 2.137550542 | 0.006964257 |
| Hispid2B012578 | ENSMUSP00000113277.1 | Tnfrsf18 | 2.136383253 | 0.034320355 |
| Hispid2B010437 | ENSMUSP00000026571.4 | Irf7 | 2.13564486 | 0.02486386 |
| Hispid2B019900 | ENSMUSP00000034602.7 | Cd3d | 2.130820237 | 0.027398093 |
| Hispid2B006740A |  |  | 2.128507417 | 0.002719612 |
| Hispid2ncA032859 |  |  | 2.111769401 | 0.023652455 |
| Hispid2B024428 | ENSMUSP00000099570.4 | Pla2g5 | 2.10975768 | 0.012109626 |
| Hispid2B003706 | ENSMUSP00000099896.1 | Cd3e | 2.100938672 | 0.010155813 |
| Hispid2B003905 |  |  | 2.090096026 | 0.020053446 |
| Hispid2B018797 |  |  | 2.085771905 | 0.047734776 |
| Hispid2B028417 |  |  | 2.085452157 | 0.005642468 |
| Hispid2B014139 | ENSMUSP00000064839.7 | Ttk | 2.078264657 | 0.011401544 |
| Hispid2B029870 |  |  | 2.07789845 | 0.025243808 |
| Hispid2B012790 |  |  | 2.069986546 | 0.025953942 |
| Hispid2B005005 | ENSMUSP00000103207.2 | Sh2d2a | 2.069957729 | 0.043847223 |
| Hispid2B018659 | ENSMUSP00000034742.6 | Ccnb2 | 2.06903489 | 0.007437105 |
| Hispid2B021210 | ENSMUSP00000143486.1 | Tex13c2 | 2.066640929 | 0.03160882 |
| Hispid2B009350 |  |  | 2.062375567 | 0.010776268 |
| Hispid2B002025 | ENSMUSP00000029456.4 | Cd2 | 2.060881528 | 0.02008298 |
| Hispid2B010789 | ENSMUSP00000005365.8 | Spc25 | 2.059044435 | 0.004818533 |
| Hispid2B006666 |  |  | 2.057476515 | 0.022608583 |
| Hispid2B019993 | ENSMUSP00000111405.1 | Foxp3 | 2.057209554 | 0.004252488 |
| Hispid2B016887 |  |  | 2.050131029 | 0.004213112 |
| Hispid2B024744 | ENSMUSP00000091073.4 | Podnl1 | 2.045431726 | 0.047464044 |
| Hispid2B016344 |  |  | 2.041633967 | 0.020053446 |
| Hispid2B022822 | ENSMUSP00000054263.8 | Hjurp | 2.033406823 | 0.018150675 |
| Hispid2B015016 | ENSMUSP00000055930.5 | Hs3st3a1 | 2.032264902 | 0.010750775 |
| Hispid2B019104 | ENSMUSP00000131126.1 | Lax1 | 2.026186687 | 0.015516244 |
| Hispid2B023577 |  |  | 2.025438152 | 0.017426687 |
| Hispid2B024210 | ENSMUSP00000055427.7 | Cdc25c | 2.019795646 | 0.018614355 |
| Hispid2B015409 |  |  | 2.018074923 | 0.036560338 |
| Hispid2B031721 | ENSMUSP00000119403.1 | Plaat3 | 2.012499562 | 0.00534572 |
| Hispid2B031517 | ENSMUSP00000038121.4 | Socs1 | 2.012372606 | 0.027901442 |
| Hispid2B025932 | ENSMUSP00000109795.2 | Crmp1 | 2.008654577 | 0.004349996 |
| Hispid2B012285 | ENSMUSP00000106953.1 | Apoa2 | 2.007982339 | 0.020949349 |
| Hispid2B014103 |  |  | 2.002785587 | 0.038512024 |
| Hispid2B023573 |  |  | 2.001202984 | 0.027398093 |
| Hispid2B000944 | ENSMUSP00000020586.6 | Slc22a4 | 0.498966123 | 0.000546027 |
| Hispid2B003600 | ENSMUSP00000049414.3 | Dusp8 | 0.498737527 | 0.00374589 |
| Hispid2B017413 | ENSMUSP00000032185.7 | Slc6a6 | 0.49837769 | 0.026186925 |
| Hispid2ncA039162 |  |  | 0.498268496 | 0.018063859 |
| Hispid2B005667 | ENSMUSP00000031985.6 | Mkrn1 | 0.498093298 | 0.003655567 |
| Hispid2B001204 | ENSMUSP00000063548.7 | Abat | 0.497990081 | 0.014103141 |
| Hispid2B017423 | ENSMUSP00000027675.7 | Pigr | 0.497867333 | 0.003655567 |
| Hispid2B010679 | ENSMUSP00000022986.6 | Fbxo32 | 0.495143255 | 0.007081426 |
| Hispid2B027017 | ENSMUSP00000134141.1 | Rfx3 | 0.494359626 | 0.01918089 |
| Hispid2ncA037889 |  |  | 0.49429655 | 0.026462679 |
| Hispid2B005643 | ENSMUSP00000042405.6 | Scn7a | 0.493632505 | 0.006492973 |
| Hispid2B029817 | ENSMUSP00000048345.6 | Hectd4 | 0.4934376 | 0.022487652 |
| Hispid2B031624 |  |  | 0.491417936 | 0.000967387 |
| Hispid2B009516 | ENSMUSP00000023832.6 | Rgn | 0.491095551 | 0.013476113 |
| Hispid2B013397 |  |  | 0.489228705 | 0.003250733 |
| Hispid2B022287 |  |  | 0.488423093 | 0.002195233 |
| Hispid2B001723 |  |  | 0.488347909 | 0.043328795 |
| Hispid2B024263 |  |  | 0.487835997 | 0.044800038 |
| Hispid2B008103 |  |  | 0.486923389 | 0.001660498 |
| Hispid2ncA033471 |  |  | 0.486762578 | 0.032793051 |
| Hispid2B011604 | ENSMUSP00000043753.7 | Ttll7 | 0.48567655 | 0.002375214 |
| Hispid2B025040 |  |  | 0.485109332 | 0.030218634 |
| Hispid2ncA032850 |  |  | 0.484614824 | 0.01753702 |
| Hispid2B027968 |  |  | 0.482425138 | 0.028727353 |
| Hispid2B010193 | ENSMUSP00000076130.5 | Plk3 | 0.481219498 | 0.006222458 |
| Hispid2B021085 | ENSMUSP00000132366.1 | Apold1 | 0.480792481 | 0.006611132 |
| Hispid2B004973 | ENSMUSP00000099071.3 | Dgki | 0.479957629 | 0.012670691 |
| Hispid2B000938 | ENSMUSP00000097527.3 | Calcrl | 0.47924293 | 0.025750925 |
| Hispid2B027929 |  |  | 0.478267566 | 0.032334048 |
| Hispid2B020231 | ENSMUSP00000028389.3 | Frzb | 0.477872153 | 0.008408483 |
| Hispid2B030040 | ENSMUSP00000029658.7 | Enpep | 0.477790676 | 0.008586152 |
| Hispid2B018765 | ENSMUSP00000081545.3 | Pappa | 0.477487572 | 0.006964257 |
| Hispid2B011553 | ENSMUSP00000048929.5 | Aox4 | 0.476076289 | 0.014211996 |
| Hispid2B014808 | ENSMUSP00000144721.1 | Wdr49 | 0.476029333 | 0.041221614 |
| Hispid2B013365 | ENSMUSP00000033721.6 | Arhgap6 | 0.475667644 | 0.047542105 |
| Hispid2B002086 |  |  | 0.474561192 | 0.022145167 |
| Hispid2B013484 | ENSMUSP00000035115.4 | Vipr1 | 0.473930624 | 0.015269437 |
| Hispid2B000848 | ENSMUSP00000038514.6 | Irs2 | 0.472026789 | 0.001768698 |
| Hispid2ncA037654 |  |  | 0.471883937 | 0.008726886 |
| Hispid2B007601 | ENSMUSP00000136302.1 | Calhm3 | 0.471733998 | 0.035995104 |
| Hispid2B008037 |  |  | 0.466289457 | 0.006879585 |
| Hispid2B018872 | ENSMUSP00000145700.1 | Fam71e1 | 0.464429127 | 0.02025488 |
| Hispid2B026610 | ENSMUSP00000103191.2 | Plxdc1 | 0.464264173 | 0.0009726 |
| Hispid2B025552 | ENSMUSP00000050680.3 | Kcna3 | 0.463584931 | 0.032214987 |
| Hispid2B015293 | ENSMUSP00000047393.5 | Bpgm | 0.461189242 | 0.004844752 |
| Hispid2B011397 | ENSMUSP00000003154.5 | Efna2 | 0.460657752 | 0.021669725 |
| Hispid2B007517 |  |  | 0.460600676 | 0.005744877 |
| Hispid2B005085 |  |  | 0.460103066 | 0.009846942 |
| Hispid2B004458 |  |  | 0.456823434 | 0.042931095 |
| Hispid2B016200 | ENSMUSP00000080791.4 | Ube2o | 0.454346276 | 0.001038701 |
| Hispid2B028644 |  |  | 0.453087294 | 0.016856372 |
| Hispid2B024960 | ENSMUSP00000149214.1 | Csrnp1 | 0.451358941 | 0.003093364 |
| Hispid2B029142 | ENSMUSP00000081867.6 | Dnah11 | 0.450376824 | 0.03546456 |
| Hispid2B002938 | ENSMUSP00000104906.1 | Slc2a13 | 0.450167796 | 0.007783024 |
| Hispid2B017010 | ENSMUSP00000020323.5 | Avpr1a | 0.449568053 | 0.004189911 |
| Hispid2B008929 | ENSMUSP00000047480.8 | Kcnab1 | 0.445753806 | 0.000980622 |
| Hispid2B008201 |  |  | 0.444829541 | 0.008218847 |
| Hispid2ncA040065 |  |  | 0.444354982 | 0.006879585 |
| Hispid2B021102 | ENSMUSP00000081044.4 | Htra4 | 0.441390477 | 0.0030558 |
| Hispid2B031504 |  |  | 0.439828221 | 0.004189911 |
| Hispid2B025994 | ENSMUSP00000020679.2 | Nipal4 | 0.435168746 | 0.006403496 |
| Hispid2B028025 | ENSMUSP00000033539.6 | F8 | 0.435142471 | 0.012030853 |
| Hispid2B016451 |  |  | 0.430513233 | 0.006158141 |
| Hispid2ncA037080 |  |  | 0.429957049 | 0.000823411 |
| Hispid2ncA039882 |  |  | 0.429679982 | 0.026163108 |
| Hispid2B011205 | ENSMUSP00000025004.6 | Adgre4 | 0.428889536 | 0.000969988 |
| Hispid2B021556 |  |  | 0.427707788 | 0.02525397 |
| Hispid2B023405 | ENSMUSP00000110102.1 | Nfkbiz | 0.426940188 | 0.00645103 |
| Hispid2B030055 | ENSMUSP00000113333.1 | Kcnt2 | 0.423786799 | 0.015454728 |
| Hispid2ncA036540 |  |  | 0.422906861 | 0.001221082 |
| Hispid2B019620 | ENSMUSP00000148417.1 | Npy1r | 0.421425805 | 0.006877546 |
| Hispid2B022048 | ENSMUSP00000116886.1 | Pcdh19 | 0.420783916 | 0.000546027 |
| Hispid2B024785 | ENSMUSP00000064823.7 | Klf2 | 0.417202197 | 0.000127991 |
| Hispid2B004779 |  |  | 0.414509763 | 0.004189911 |
| Hispid2B008032 | ENSMUSP00000032958.7 | Ucp3 | 0.414258777 | 0.001353022 |
| Hispid2B011347 |  |  | 0.413729359 | 0.015454728 |
| Hispid2B001903 |  |  | 0.409571805 | 0.01456849 |
| Hispid2B006683 | ENSMUSP00000142576.1 | Magi2 | 0.409315835 | 0.004189911 |
| Hispid2B010206 | ENSMUSP00000062702.3 | Fam171b | 0.404859166 | 0.003434767 |
| Hispid2B026686 |  |  | 0.404787402 | 0.025953942 |
| Hispid2B022433 | ENSMUSP00000029946.7 | Rragd | 0.403648915 | 0.000112829 |
| Hispid2B031447 | ENSMUSP00000021458.6 | Sptb | 0.402870136 | 0.014693917 |
| Hispid2B025682 | ENSMUSP00000128063.1 | Sec14l5 | 0.402506011 | 0.008226031 |
| Hispid2B030438 |  |  | 0.40244337 | 0.000329054 |
| Hispid2B019289 | ENSMUSP00000118772.1 | Treml4 | 0.402282251 | 0.012858457 |
| Hispid2B025743 | ENSMUSP00000124022.2 | Pappa2 | 0.400983734 | 0.012077412 |
| Hispid2B000560 | ENSMUSP00000043416.6 | Rtp1 | 0.400267541 | 0.004607206 |
| Hispid2B030419 | ENSMUSP00000153142.1 | Nek10 | 0.400061906 | 0.018810356 |
| Hispid2B013110 | ENSMUSP00000028166.2 | Nr4a2 | 0.398766799 | 0.004515466 |
| Hispid2ncA033008 |  |  | 0.397790296 | 0.02025488 |
| Hispid2B006677 | ENSMUSP00000036227.5 | St6galnac5 | 0.396120815 | 0.024291785 |
| Hispid2B006486 | ENSMUSP00000121817.1 | Nfe2 | 0.39041292 | 0.004213112 |
| Hispid2B003402 | ENSMUSP00000030673.6 | Csf3r | 0.389837303 | 0.017635257 |
| Hispid2B018515 | ENSMUSP00000020171.5 | Ccn2 | 0.389070006 | 0.0002711 |
| Hispid2B010320 | ENSMUSP00000075488.5 | Srrm4 | 0.387722237 | 0.025628907 |
| Hispid2ncA038236 |  |  | 0.387312506 | 0.029402957 |
| Hispid2B012617 | ENSMUSP00000034597.7 | Tmprss13 | 0.385905493 | 0.012109626 |
| Hispid2B031166 | ENSMUSP00000135855.3 | Nptxr | 0.385040569 | 0.003434767 |
| Hispid2B011280 | ENSMUSP00000150887.1 | Zbtb16 | 0.382500356 | 0.02486386 |
| Hispid2B014515 | ENSMUSP00000137298.1 | Tdrd5 | 0.381592862 | 0.040934475 |
| Hispid2B020032 | ENSMUSP00000046191.7 | Elmod1 | 0.380342186 | 0.001894475 |
| Hispid2B018694 | ENSMUSP00000026045.7 | Col17a1 | 0.379627533 | 0.023652455 |
| Hispid2B002568 | ENSMUSP00000099063.2 | Upp1 | 0.379294047 | 0.015281482 |
| Hispid2ncA039910 |  |  | 0.3780226 | 0.04843157 |
| Hispid2ncA038887 |  |  | 0.377836797 | 0.022841705 |
| Hispid2B004884 | ENSMUSP00000100615.2 | Nrarp | 0.377633447 | 0.004375586 |
| Hispid2B017574 | ENSMUSP00000130551.1 | Hspb9 | 0.375312423 | 0.046184913 |
| Hispid2B014193 | ENSMUSP00000112991.1 | Pde4d | 0.374558974 | 0.009445718 |
| Hispid2B020796 | ENSMUSP00000143344.1 | Celf3 | 0.369103663 | 0.045666393 |
| Hispid2ncA039629 |  |  | 0.367795912 | 0.048480612 |
| Hispid2ncA040086 |  |  | 0.366513503 | 0.048005795 |
| Hispid2B028027 | ENSMUSP00000029846.3 | Ccn1 | 0.365977964 | 0.032120695 |
| Hispid2ncA038840 |  |  | 0.365483259 | 0.03375643 |
| Hispid2B003572 |  |  | 0.362035167 | 0.018828851 |
| Hispid2B030339 |  |  | 0.361623261 | 0.00645103 |
| Hispid2B022165 | ENSMUSP00000087805.4 | Muc15 | 0.359352209 | 0.03556579 |
| Hispid2B003099 |  |  | 0.354035987 | 0.034546954 |
| Hispid2B020462 | ENSMUSP00000039586.4 | Serpine1 | 0.352891556 | 0.015598784 |
| Hispid2ncA039771 |  |  | 0.352491179 | 0.029292194 |
| Hispid2B031192 | ENSMUSP00000080533.3 | Tmeff2 | 0.350829865 | 0.036829152 |
| Hispid2B031135 |  |  | 0.350018161 | 0.04731319 |
| Hispid2B009659 |  |  | 0.348166024 | 0.020367792 |
| Hispid2B020537 | ENSMUSP00000112769.1 | Fam107a | 0.341136632 | 0.004607206 |
| Hispid2B010659 |  |  | 0.341121879 | 1.03E-05 |
| Hispid2B022677 | ENSMUSP00000021932.5 | Drd1 | 0.338718675 | 0.016675165 |
| Hispid2B002493 | ENSMUSP00000029141.5 | Mmp24 | 0.338562233 | 0.01165596 |
| Hispid2B005454 | ENSMUSP00000109403.1 | Itgb2l | 0.336524006 | 0.038425206 |
| Hispid2B018661 | ENSMUSP00000042183.6 | Ttc36 | 0.335138889 | 0.027345838 |
| Hispid2B010334 |  |  | 0.334881631 | 0.000100008 |
| Hispid2B023161 | ENSMUSP00000102524.1 | Pde4b | 0.334880286 | 0.000917328 |
| Hispid2B019737 | ENSMUSP00000027422.5 | Slc16a14 | 0.334293935 | 0.033920399 |
| Hispid2B026173 |  |  | 0.333729492 | 0.014116608 |
| Hispid2B030101 | ENSMUSP00000052894.7 | Fpr1 | 0.331825966 | 0.006964257 |
| Hispid2B016222 | ENSMUSP00000052648.7 | Ar | 0.331672359 | 0.014454561 |
| Hispid2B000167 |  |  | 0.330680819 | 0.007778038 |
| Hispid2B027271 | ENSMUSP00000137374.1 | Pou3f1 | 0.327272803 | 0.00359841 |
| Hispid2B026863 | ENSMUSP00000116779.1 | Fam131b | 0.320038101 | 0.016698377 |
| Hispid2B007623 |  |  | 0.318651669 | 0.010835019 |
| Hispid2B007108 | ENSMUSP00000048904.2 | Hsf4 | 0.317385832 | 0.007070127 |
| Hispid2B007071 | ENSMUSP00000103245.2 | Klf4 | 0.313886478 | 9.08E-05 |
| Hispid2B026034 | ENSMUSP00000128215.1 | Treml2 | 0.313376761 | 0.044521674 |
| Hispid2ncA037493 |  |  | 0.312278357 | 0.014103141 |
| Hispid2B030954 |  |  | 0.307099763 | 0.001056251 |
| Hispid2ncA033016 |  |  | 0.303404702 | 0.043847921 |
| Hispid2B010738 | ENSMUSP00000065825.7 | Rgs17 | 0.301717213 | 0.039775135 |
| Hispid2B025516 | ENSMUSP00000021028.4 | Itgb3 | 0.301673564 | 0.012109626 |
| Hispid2B026165 |  |  | 0.300478778 | 0.019624688 |
| Hispid2ncA039683 |  |  | 0.298768014 | 0.014116608 |
| Hispid2B027038 |  |  | 0.297773199 | 0.023652455 |
| Hispid2B012187 | ENSMUSP00000035417.6 | Pklr | 0.296479609 | 0.033649661 |
| Hispid2B012422 | ENSMUSP00000032573.6 | Pglyrp1 | 0.292633336 | 0.02852677 |
| Hispid2B019189 |  |  | 0.291988983 | 0.000773587 |
| Hispid2B017477 |  |  | 0.29176092 | 0.0030272 |
| Hispid2B027380 |  |  | 0.289743537 | 0.000546027 |
| Hispid2B026995 |  |  | 0.287918328 | 0.000969988 |
| Hispid2B022626 | ENSMUSP00000066040.6 | Alas2 | 0.287643376 | 0.00067019 |
| Hispid2B011261 |  |  | 0.284266242 | 0.0009726 |
| Hispid2ncA036550 |  |  | 0.283347023 | 0.013970675 |
| Hispid2B024445 | ENSMUSP00000007340.2 | Atp12a | 0.283182188 | 0.032334048 |
| Hispid2B001058 |  |  | 0.282100955 | 0.017197677 |
| Hispid2ncA036811 |  |  | 0.280751275 | 0.018264193 |
| Hispid2B013286 |  |  | 0.279904398 | 0.027398093 |
| Hispid2B023462 | ENSMUSP00000063279.6 | Btnl10 | 0.277311457 | 0.030097045 |
| Hispid2B023647 | ENSMUSP00000067837.2 | Bpi | 0.277022813 | 0.005000534 |
| Hispid2B002504 |  |  | 0.276498651 | 0.043993718 |
| Hispid2B018484 | ENSMUSP00000128052.1 | Gfi1b | 0.275658494 | 0.01456849 |
| Hispid2B004325 | ENSMUSP00000015583.1 | Ctsg | 0.275164532 | 0.018847985 |
| Hispid2B013327 | ENSMUSP00000153491.1 | Egr3 | 0.27144505 | 0.034988518 |
| Hispid2B020592 | ENSMUSP00000126008.1 | Cst12 | 0.270613811 | 0.047330628 |
| Hispid2B001751 |  |  | 0.266451643 | 0.031033104 |
| Hispid2B005319 | ENSMUSP00000003640.2 | Fosb | 0.262854417 | 0.032214987 |
| Hispid2B012333 | ENSMUSP00000032262.7 | Clec1b | 0.26258834 | 0.026513793 |
| Hispid2B028969 | ENSMUSP00000032476.4 | Slc2a3 | 0.261727017 | 0.027777527 |
| Hispid2B014023 | ENSMUSP00000025685.6 | Lipm | 0.260438876 | 0.004077968 |
| Hispid2B003595 |  |  | 0.257427726 | 0.034883936 |
| Hispid2B004130 | ENSMUSP00000031894.6 | Clcn1 | 0.257399031 | 0.007906589 |
| Hispid2B002272 | ENSMUSP00000023779.6 | Nr4a1 | 0.254575581 | 0.002719612 |
| Hispid2B006311 | ENSMUSP00000044245.6 | Prdm14 | 0.254211948 | 0.025527297 |
| Hispid2B016442 | ENSMUSP00000034453.4 | Acta1 | 0.253641483 | 0.000407065 |
| Hispid2B018252 | ENSMUSP00000141553.1 | Tnr | 0.253333861 | 0.000980622 |
| Hispid2B017238 |  |  | 0.253178338 | 0.025953942 |
| Hispid2ncA036763 |  |  | 0.251888211 | 0.002040649 |
| Hispid2B013879 | ENSMUSP00000033512.4 | Slc38a5 | 0.246728964 | 0.005196663 |
| Hispid2B013729 |  |  | 0.246312663 | 0.034269259 |
| Hispid2B012890 | ENSMUSP00000126029.1 | Cacna2d2 | 0.245580148 | 0.038394851 |
| Hispid2B018226 |  |  | 0.244879905 | 0.034269259 |
| Hispid2B013088 |  |  | 0.244565191 | 0.012109626 |
| Hispid2B000852 |  |  | 0.240553026 | 0.033043705 |
| Hispid2B027014 | ENSMUSP00000113864.1 | Glt1d1 | 0.23774712 | 0.0373441 |
| Hispid2B022738 | ENSMUSP00000139132.1 | Slfn14 | 0.235625127 | 0.030567155 |
| Hispid2B007173 | ENSMUSP00000070842.5 | S100a9 | 0.234193248 | 0.042642848 |
| Hispid2B007929 |  |  | 0.228145955 | 0.01911199 |
| Hispid2B017620 | ENSMUSP00000112338.2 | Prr16 | 0.226523087 | 0.008322146 |
| Hispid2ncA038378 |  |  | 0.224087221 | 0.010433601 |
| Hispid2ncA037570 |  |  | 0.223759572 | 0.004213112 |
| Hispid2B025427 | ENSMUSP00000148015.1 | Cnih3 | 0.223195716 | 0.010988842 |
| Hispid2B005646 | ENSMUSP00000026381.6 | Padi4 | 0.222136007 | 0.010613501 |
| Hispid2B027180 | ENSMUSP00000027243.7 | Il1r2 | 0.220152574 | 0.019349067 |
| Hispid2ncA036683 |  |  | 0.216935776 | 0.00670022 |
| Hispid2B002419 | ENSMUSP00000051392.3 | Rln3 | 0.216514729 | 0.032598096 |
| Hispid2B011652 | ENSMUSP00000045315.7 | Mfsd2b | 0.20612658 | 0.040927587 |
| Hispid2B022098 | ENSMUSP00000126067.1 | Snca | 0.204569986 | 0.001611968 |
| Hispid2B015104 |  |  | 0.203426043 | 0.027552431 |
| Hispid2B001013 |  |  | 0.202988808 | 0.009999659 |
| Hispid2B008046 | ENSMUSP00000043281.7 | Dnah1 | 0.202342085 | 0.004607206 |
| Hispid2ncA039944 |  |  | 0.196730104 | 0.034158485 |
| Hispid2B002669 | ENSMUSP00000102921.1 | Crnn | 0.196022449 | 0.034972095 |
| Hispid2B006603 |  |  | 0.196005386 | 0.043448106 |
| Hispid2B024125 |  |  | 0.195197511 | 0.005832266 |
| Hispid2B025051 |  |  | 0.189209205 | 0.011655946 |
| Hispid2B029107 |  |  | 0.188491783 | 0.026163108 |
| Hispid2B017347 | ENSMUSP00000052585.3 | Tprg | 0.180517132 | 0.012534008 |
| Hispid2B016542 | ENSMUSP00000057563.6 | Gp1ba | 0.175400827 | 0.012109626 |
| Hispid2B002083 | ENSMUSP00000066383.2 | Hemgn | 0.174427061 | 0.006879585 |
| Hispid2B028323 | ENSMUSP00000063136.4 | Htr1f | 0.173750575 | 0.033971706 |
| Hispid2B015063 |  |  | 0.169649441 | 0.043734378 |
| Hispid2B003874 | ENSMUSP00000099375.3 | Itga2b | 0.166368166 | 0.008463662 |
| Hispid2B016294 |  |  | 0.165788389 | 0.027175729 |
| Hispid2ncA037993 |  |  | 0.163407584 | 0.003302375 |
| Hispid2B000437 |  |  | 0.159294885 | 0.012183033 |
| Hispid2B020828 |  |  | 0.158499545 | 0.025527297 |
| Hispid2ncA038420 |  |  | 0.154347602 | 0.00495467 |
| Hispid2B007922 |  |  | 0.147943746 | 0.014274987 |
| Hispid2B030927 | ENSMUSP00000027372.7 | Cxcr2 | 0.146951533 | 0.012490974 |
| Hispid2B008185 |  |  | 0.146162178 | 0.02008298 |
| Hispid2B008373 | ENSMUSP00000129116.1 | Sis | 0.142242146 | 0.030384676 |
| Hispid2ncA037809 |  |  | 0.135623857 | 0.003511832 |
| Hispid2B014302 |  |  | 0.134281152 | 0.01753702 |
| Hispid2B011571 |  |  | 0.13340863 | 0.029954746 |
| Hispid2B031586 |  |  | 0.132580849 | 0.033492279 |
| Hispid2ncA037595 |  |  | 0.129475521 | 0.022574826 |
| Hispid2B023461 | ENSMUSP00000101938.1 | 6030468B19Rik | 0.128288993 | 0.006217166 |
| Hispid2B017039 |  |  | 0.12455919 | 0.040066887 |
| Hispid2B027596 |  |  | 0.122675082 | 0.029767993 |
| Hispid2B004012 | ENSMUSP00000031899.8 | Kel | 0.122211639 | 0.001021622 |
| Hispid2B031559 |  |  | 0.121001075 | 0.025814395 |
| Hispid2B012627 |  |  | 0.114839829 | 0.034450236 |
| Hispid2B028114 | ENSMUSP00000064366.7 | Klf1 | 0.113724384 | 0.010155813 |
| Hispid2B007861 |  |  | 0.111463135 | 0.030031603 |
| Hispid2B018534 |  |  | 0.109288714 | 0.02088917 |
| Hispid2ncA037733 |  |  | 0.108622018 | 0.015454728 |
| Hispid2B014148 |  |  | 0.108204081 | 0.037305576 |
| Hispid2B028339 |  |  | 0.103557241 | 0.004166186 |
| Hispid2ncA039587 |  |  | 0.103205097 | 0.001021622 |
| Hispid2B019978 | ENSMUSP00000024721.7 | Rhag | 0.102185808 | 0.007783024 |
| Hispid2B018645 |  |  | 0.100536008 | 0.020053446 |
| Hispid2B010147 |  |  | 0.099789575 | 0.004596999 |
| Hispid2B004886 |  |  | 0.099763182 | 0.032190095 |
| Hispid2B025475 |  |  | 0.096413056 | 0.044842273 |
| Hispid2B019867 | ENSMUSP00000006749.9 | Slc4a1 | 0.091369226 | 0.001985246 |
| Hispid2B021951 | ENSMUSP00000134201.1 | Brsk2 | 0.090726745 | 0.00670022 |
| Hispid2B011024 |  |  | 0.089336064 | 0.015999508 |
| Hispid2B028881 | ENSMUSP00000027639.1 | Marco | 0.081087994 | 0.008724279 |
| Hispid2B001949 |  |  | 0.077174451 | 0.015454728 |
| Hispid2B021186 | ENSMUSP00000031319.6 | Ppbp | 0.073815093 | 0.017442648 |
| Hispid2B021187 | ENSMUSP00000027817.7 | Spta1 | 0.070737071 | 0.002997628 |
| Hispid2B031388 |  |  | 0.066572402 | 0.010063956 |
| Hispid2B016515 | ENSMUSP00000046380.6 | Ly6g6f | 0.064940599 | 0.01456849 |
| Hispid2B015701 |  |  | 0.064029019 | 0.022161422 |
| Hispid2B012089 | ENSMUSP00000104453.1 | Gm12253 | 0.054829795 | 0.003302375 |
| Hispid2B022469 | ENSMUSP00000123476.1 | Gm4846 | 0.049945515 | 1.03E-05 |
| Hispid2B013705 | ENSMUSP00000027151.5 | Myl1 | 0.047109998 | 1.03E-05 |
| Hispid2B023715 | ENSMUSP00000153300.1 | Treml1 | 0.041972683 | 0.019823676 |
| Hispid2B031128 | ENSMUSP00000017881.2 | Mmp9 | 0.041576462 | 0.021340274 |
| Hispid2B014928 | ENSMUSP00000137926.1 | Car1 | 0.036847375 | 0.006877546 |
| Hispid2ncA037347 |  |  | 0.027920736 | 0.000756569 |
| Hispid2B031358 |  |  | 0.011382869 | 0.032622033 |
| Hispid2B031835 |  |  | 0.010635183 | 0.024013848 |
| Hispid2B014537 |  |  | 0.007683642 | 0.033039925 |

**Supplemental Table 5. Differentially expressed genes between adult and geriatric cotton rats at day 4 post-RSV infection with FC >2 or <0.5 and q-values of <0.05.** Green cells represent genes with higher expression in adults. Peach cells represent genes with higher expression in geriatrics.
