# Supplementary material for: Reduced inflammatory and Th1 transcriptional profiles in geriatric versus adult cotton rats infected with respiratory syncytial virus
Source: PLoS Pathog. 2026 Jul 9;22(7):e1014323. doi: 10.1371/journal.ppat.1014323 (PMC13349118; doi:10.1371/journal.ppat.1014323)
Supplement: Attachment — Submitted filename: Response to Reviewers.docx [file ppat.1014323.s007.docx]

Dear editor and reviewers,

The reviewers of our manuscript “Reduced inflammatory and Th1 transcriptional profiles in geriatric versus adult cotton rats infected with respiratory syncytial virus” provided thoughtful feedback that we believe resulted in meaningful improvements to the article. Below is a point-by-point list of issues raised by the reviewers, followed by the ways in which we have addressed them.

---

**Reviewer 1**

- The results are reported sometimes in the past tense and other times in present tense. Perhaps normalize everything to the past tense?

We have reviewed the tense of our results and discussion, editing to more consistently refer to our findings consistently in the past tense.

- The sentence on lines 134-137 is somewhat confusing and should probably be reworded.

The previous sentence read “Homogenization of lung was performed the same day as collection, following transportation at room temperature in RNAprotect (QIAGEN).” We restructured this sentence and gave a specific time interval for exposure to RNAprotect; the sentence now reads “Following euthanasia, lungs were placed in RNAprotect (QIAGEN) at room temperature for 30 minutes,” followed by detailed description of the homogenization procedure.

- The sentence on lines 121-122 should be revised with more details provided on what was actually done.

The TCID50 assay description had incorrectly omitted the number of wash steps with the media added afterward. This has been corrected.

- Fig.1 legend. Replace “in whole lung” with “in the lung”, as technically only the left lung was used for analysis. The same should be done for the legend for Fig. 2.

This change has been made.

- Perhaps provide an explanation for why IL-12p35 and not IL-12b was chosen for the digital PCR analysis?

The sentence “IL-12p35 encodes the IL-12-specific alpha subunit, while IL-12B is a common subunit of both IL-12 and IL-23; as such IL-12p35 was measured as a more specific measure of IL-12 expression” has been added to the ddPCR results section.

- The definition of “geriatric” cotton rats is different in different parts of the manuscript. In “Methods” it is listed as >8 months old, in the legend to Fig.5 as “>12 months of age”. Making it more consistent would help the reader.

While we define geriatric cotton rats as >8 months of age as described in the Methods section, we feel it is important to state that the cohort in the experiment for Figure 5 were animals over 12 months of age. To clarify that “>12 months of age” refers to that cohort and not to a definition of geriatric cotton rats, we amended the parenthetical definition to read “(>12 months of age for this cohort)”.

- On line 379 the word “detectable” should be added before “IL-4”. The word “detectable” (or “elevated) should also be considered on line 380 before the word “secretion”.

This change has been made.

**Reviewer 2**

- The authors should include a figure showing the comparison of the DEGS adult vs geriatric animals and list all the DEGS as a supplementary figure. This figure should be relatively easy to generate, as the data already exists.

We have added Fig. 2, a Venn diagram showing the numbers of DEGs in adult vs geriatric animals at days 0, 1, and 4 post-RSV infection. We also added lists of all DEGs at days 0, 1, and 4 post-infection as Supplemental Tables 3, 4, and 5, respectively.

**---**

We have also added sections for Author Summary (following the Abstract) and List of Legends for Supplemental Information (following References) in accordance with PLOS Pathogens requirements.

We thank you for your attentive consideration of our manuscript and look forward to your review.

Sincerely,

Jonathan Miller, DVM, PhD, DACVP

Assistant Professor of Veterinary Anatomic Pathology

Department of Veterinary Microbiology and Pathology

College of Veterinary Medicine

Washington State University

jonathan.miller2@wsu.edu
